# The Structural Biology of T‐Cell Antigen Detection at Close Contacts

**DOI:** 10.1111/imr.70014

**Published:** 2025-04-03

**Authors:** Yuan Lui, João Ferreira Fernandes, Mai T. Vuong, Sumana Sharma, Ana Mafalda Santos, Simon J. Davis

**Affiliations:** ^1^ Weatherall Institute of Molecular Medicine, Radcliffe Department of Medicine, John Radcliffe Hospital University of Oxford Oxford UK; ^2^ Medical Research Council Translational Immune Discovery Unit, John Radcliffe Hospital University of Oxford Oxford UK

**Keywords:** antigen recognition, close contacts, receptor triggering, structural biology, T‐cell signaling

## Abstract

T cells physically interrogate their targets using tiny membrane protrusions called microvilli, forming junctions ~400 nm in diameter and ~ 15 nm deep, referred to as “close contacts”. These contacts, which are stabilized by the binding of the small adhesion protein CD2 to its ligand, CD58 and locally exclude large proteins such as the phosphatase CD45, are the sites of antigen recognition by the T‐cell receptor (TCR) and very early signaling by T cells. With our collaborators, we have characterized the molecular structures of several of the key proteins mediating these early events: i.e., CD2 and its ligands, CD45, the αβ‐ and γδ‐TCRs, and the accessory proteins CD28, CTLA‐4, and PD‐1. Here, we review our structural work and the insights it offers into the early events underpinning T‐cell responsiveness that take place in the confined space of the close contact. We reflect on the crucial roles that the structural organization and dimensions of these proteins are likely to have in determining the sequence of events leading to antigen recognition at close contacts and consider the general implications of the structural work for explanations of how immune receptor signaling is initiated.

## 1 Introduction

To perform their immune functions, T cells need to be highly tactile [1, 2]. They protect us from infections and tumors by being able to sense the physical presence, on prospective target cells, of antigenic peptides bound to major histocompatibility complex molecules (pMHC) using specialized structures, that is, T‐cell receptors (TCRs) [3, 4]. Encounters with pMHC result in TCR phosphorylation, the first step in T‐cell activation. Exactly how the receptor becomes phosphorylated is uncertain [5, 6]. Our proposal, known as the “kinetic‐segregation” (KS) model [7, 8], supposes that the net level of TCR phosphorylation by kinases, such as Lck, increases when large receptor‐type protein tyrosine phosphatases (RPTPs), such as CD45, are sterically excluded from sites of TCR/pMHC binding, preventing them from reversing the constitutive phosphorylation of the receptor. Incipient phosphorylation of the receptor is likely to be amplified by the kinase‐bearing co‐receptors, CD4 and CD8 [9, 10, 11], allowing recruitment and activation of the cytosolic kinase, ZAP70 (reviewed in [12]). ZAP70 phosphorylates the linker for activation of T cells, which in turn forms two‐dimensional condensates controlling downstream signaling [13, 14]. TCR‐derived signals are integrated with those from “accessory” receptors, for example, the costimulatory protein CD28, which amplifies TCR signaling, and the immune checkpoints, CTLA‐4 and PD‐1, which suppress it (reviewed in [15, 16]).

The modern view of the structure of the mammalian cell surface is that it comprises two molecular layers [17, 18]. Adjacent to the cell membrane, the first layer consists of “communication devices” that is, receptors extending up to 20–30 nm from the cell surface that bind cognate ligands secreted by or expressed on the surfaces of other cells. The communication devices of T cells include, for example, the TCR, CD28, and PD‐1. The second layer, known as the “glycocalyx” is formed of dense arrays of highly‐expressed, negatively charged, and heavily‐glycosylated proteins and polysaccharides that extend beyond the first layer, creating a physical barrier to cell contact [17]. The glycocalyx of nucleated cells of hematopoietic origin is comprised of CD45 and the mucin‐like glycoprotein, CD43 [19, 20].

**FIGURE 1:**
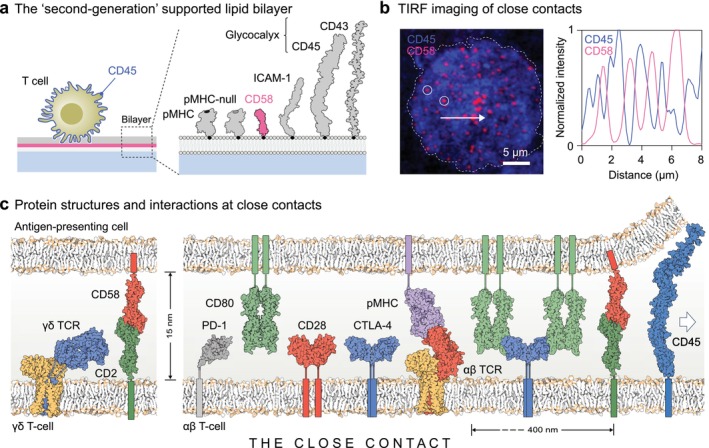
Close contacts. (a) 'Second‐generation' glass‐supported lipid bilayers present histidine‐tagged, soluble forms of the ECDs of agonistic and 'null' pMHC, the fluorescently‐tagged ligand of CD2, that is, CD58, ICAM‐1, and the two major glycocalyx elements of the antigen‐presenting cell surface, CD45 and CD43, via nickellated lipids. (b) T cells, labeled with fluorescent anti‐CD45 antibody (blue), punch through the glycocalyx barrier on the bilayer and form CD2‐mediated close contacts marked by the local accumulation of CD58 (magenta) in 400 nm‐wide puncta visible using total internal reflection fluorescence (TIRF) microscopy (two such puncta are circled, *left*). The intensity line profile (*right*) showing the local exclusion of CD45 at regions of CD58 accumulation, is taken along the line indicated by the white arrow. (c) Depictions of the structures and interactions of proteins discussed in this review, prepared using the program Illustrate [21]; the TCR complexes were positioned using membrane coordinates from the Orientations of Proteins in Membranes database [22] (using PDB: 7phr).

We have studied how T cells overcome glycocalyx barriers, using supported lipid bilayer‐based models of the antigen‐presenting cell surface presenting the extracellular domains (ECDs) of the glycocalyx elements CD43 and CD45, the ligands of small (CD2) and large (LFA‐1) adhesive proteins, that is, CD58 and ICAM‐1, respectively, and both null and model agonistic TCR ligands (Figure 1a) [23]. We found that T cells use very narrow surface protrusions called microvilli to ‘punch’ through glycocalyx barriers, forming numerous tiny (400 nm‐wide) “close contacts” visualized via the accumulation of CD58 (Figure 1b) [23]. Because they are stabilized by the binding of CD2 to CD58 [23], close contacts are likely ~15 nm deep. Close contacts are sites of antigen detection and signaling, as is indicated by their accumulation of agonistic pMHC and their recruitment of ZAP70 [23]. The first sign of productive signaling, occurring within seconds, is that the cells spread on the bilayer, forming more close contacts; in the absence of the TCR, just a few close contacts form, and the cells do not spread. ICAM‐1 is excluded from the close contacts but is required during the spreading stage [23], presumably when LFA‐1 adopts a high‐affinity conformation [24]. If the contacts are made to be artificially large (i.e., > 1 μm), TCR discrimination is lost [23], as predicted by computational models of T‐cell signaling based on the KS model [25, 26]. Close contacts are likely, therefore, to be sites of early decision‐making by T cells.

Working closely with structural biologists over many years, we have characterized the molecular structures of key actors in these early signaling events: that is, the small adhesion protein CD2 [27, 28] and its ligands CD58 [29] and CD48 [30], the large phosphatase CD45 [31], αβ‐ [32] and γδ‐ [33] TCRs, and the accessory proteins, CD28 [34] and CTLA‐4 [35, 36] and their ligand CD80 [37], and PD‐1 [38]. This was made possible by first solving the ‘glycosylation problem’, which is that whereas the folding of glycoproteins is often glycosylation‐dependent (reviewed in [39]), the chemical and conformational heterogeneity of their N‐glycans frequently inhibits their crystallization (see, e.g., [40, 41]). We established several methods for blocking N‐glycan processing during the production of glycoproteins in mammalian cells, allowing removal of the glycans with endoglycosidases after the glycoproteins are purified [42, 43, 44]. Here, we review our structural work and consider how the detailed structures of these proteins contribute to antigen recognition and signaling by T cells in the confined space of the close contact. The proteins considered here are presented in the likely temporal order of their contribution to antigen recognition. An overview of the structures is given in Figure 1c. Early stages of this work have been reviewed previously [45, 46].

## 2 CD2 and Its Ligands

CD2 belongs to a family of cell surface proteins forming a subgroup of the immunoglobulin (Ig) superfamily [47]. Human CD2 and its ligand, CD58, were the first heterophilic cell adhesion molecules to be identified [48, 49], and analyses of rat CD2/ligand interactions using surface plasmon resonance‐based technology were the first to suggest that protein interactions at cell surfaces would typically have very fast off‐rates [50, 51]. Human CD2 is expressed by T‐ and NK cells and, by binding to the broadly‐expressed CD58, contributes to antiviral responses, autoimmunity, transplant rejection, and tumor immune evasion [52], likely by setting cellular activation thresholds [53, 54]. In mice and rats, CD2 binds a different but related protein, CD48 [55]. CD48 also binds to a second related protein, CD244 [56], that triggers the killing of CD48‐expressing targets by NK cells [57].

### 2.1 CD2

**FIGURE 2:**
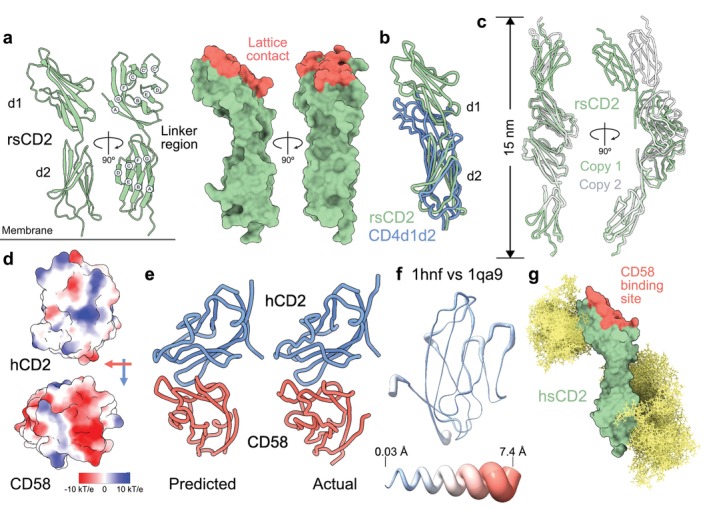
CD2. (a) Ribbon and surface‐rendered views of the rat CD2 ECD (rsCD2; PDB: 1hng) showing the Ig superfamily domain organization of the protein (*left*), and the location of residues mediating the copy 1 head‐to‐head lattice contact expected to mimic CD2/ligand interactions (red; *right*). (b) Domain 2 superposition of rsCD2 and human CD4d1d2 (PDB: 1cdh) showing the lengthening effect of the conserved linker region of CD2. (c) The two versions of the head‐to‐head lattice contact observed for the two crystallographically independent copies of the CD2 ECD in the rat sCD2 crystals. (d) The electrostatic potential calculated at neutral pH projected onto the ligand‐binding surfaces of human (h) CD2 (PDB: 1hnf; *upper*) and CD58 (PDB: 1ccz; *lower*). Blue corresponds to positive potential, white neutral, and red negative potential, contoured at ±10 kT/e. The directions of the C strands of each domain are represented by the blue (CD2) and red (CD58) arrows (relative to CD2, the CD58 ligand‐binding domain is rotated 90° clockwise in the plane of the page). (e) The structure of the CD2/CD58 d1 complex, predicted by superimposing the ligand‐binding domains of human CD2 and CD58 onto the head‐to‐head lattice contact observed in crystals of human sCD2, is shown alongside the actual structure of the complex (PDB: 1qa9), determined subsequently [60]. (f) Structural differences between the CD58‐complexed and apo forms of hCD2 d1 (*top*). Thickness of the putty cartoon representation reflects the distance between equivalent Cα atoms following superposition of the structures. The apo form of hCD2 d1 is shown; the model helix (*lower*) scales the differences. (g) Glycosylation of CD2. Fifty N‐linked glycan conformers, depicted as yellow sticks and corresponding to NeuNAc‐Gal‐(GlcNAc)_2_Man_3_(Fuc)(GlcNAc)_2_, were modeled at each of the glycosylation sites occupied with sugar in the hsCD2 crystals, using GlycoSHIELD [61]. The view shown is equivalent to that in the ribbon representation of rsCD2 in Figure 1a, *left*. The CD58 binding site of human CD2 is indicated (red).

Rat soluble (s) CD2 was the test case demonstrating the utility of N‐glycan engineering for enhancing glycoprotein crystallization [42], yielding the first structure of the ECD of a cell adhesion molecule [27]. The ECD comprises two domains with Ig superfamily folds, as had been expected [58], connected by a highly conserved “linker” [59] (Figure 2a). In the linker, the polypeptide continues in an extended conformation for four residues beyond β‐strand G of domain (d) 1 through to β‐strand A of d2. In this way, it adds ~15 Å to the length of the protein and loosens its overall structure versus an otherwise similar arrangement of N‐terminal domains in CD4 (Figure 2b). The linker positions the d1 GFCC'C″ β‐sheet, identified as the ligand‐binding site by mutagenesis of human CD2 [62, 63, 64], ~75 Å above the cell surface, close‐to‐parallel with the membrane, likely favoring head‐to‐head *in trans* interactions as cell contact is initiated (Figure 2a). A conserved glycosylation site at the base of d2 of CD2 (and likely CD58 [60]) may hold the protein ‘upright’ at the cell surface. Substantial ‘hinge‐bending’ resulting from small changes in main‐chain torsion angles in the linker could also favor binding [27].

A “head‐to‐head” contact in the crystals notable for being especially large (burying 650–690 Å^2^/molecule), suggested a plausible model of CD2/ligand interactions, comprising a complex of ~15 nm (Figure 2a,c). The ligand‐binding d1 GFCC'C″ β‐sheets of each of two copies of the rat CD2 ECD in the crystal lattice formed the contact, which was seen again in crystals of the human CD2 ECD despite their distinct space groups and the substantial compositional differences of their GFCC'C″ β‐sheets [28]. The GFCC'C″ β‐sheets of the two homologues are unusual as sites of protein–protein recognition in being both flat and highly charged (Figure 2d, upper). Alanine substitutions and the effects of ionic strength on binding indicated that two aromatics and one aliphatic residue in the GFCC'C″ β‐sheet provide all the energy needed for CD2 to bind weakly to its ligands (*K*_D_ = 60–90 μM [50]) and that eight charged or polar residues confer specificity without compromising the low affinity [65]. This is because the favorable effects of charged‐residue interactions at protein interfaces are offset by the energetic costs of de‐solvating the charged residues [66]. For more typical protein interactions, both specificity and affinity rely on the surface‐shape complementarity of relatively hydrophobic surfaces [67].

### 2.2 The Ligands, CD58 and CD48

Structural analysis of the human and rat CD2 ligands, CD58 and CD48, respectively, strengthened these arguments. The ligand‐binding domain of CD58, crystallized as a chimera with d2 of rat CD2, was broadly similar to that of human CD2 [29]. It had standard Ig superfamily V‐set AGFCC'C″:DEB domain topology, but the head‐to‐head lattice contact characteristic of the CD2 ECD crystals [27, 28] was absent. The CD2‐binding site of CD58, comprised also of its d1 GFCC'C″ β‐sheet [68, 69], had surface depressions rather than being flat, resulting in it having no shape complementarity with the binding site of CD2. The AGFCC'C″ β‐sheet was notably acidic, however (Figure 2d, lower [29]), complementing the basic CD58‐binding site of CD2. Docking CD58 and CD2 in the head‐to‐head arrangement observed in CD2 ECD crystals produced a high degree of electrostatic complementarity at the interface [29], leading us to predict that this is how they interact, as was subsequently confirmed when the actual structure of the CD2/CD58 complex was determined (Figure 2e) [60]. The structure of CD48 d1 [30] is similar to that of d1 of CD58. It differs in that, although it is also highly charged, the ligand‐binding GFCC'C″ β‐sheet of CD48 is much flatter. Modeling the interactions of rat CD2 and CD48 required a ∼7° rotation of CD48 and a ∼3 Å translation versus the arrangement observed in the CD2/CD58 complex [60] to maximize electrostatic complementarity [30]. Moreover, whereas human CD2/CD58 binding must surmount a significant entropic barrier, the rat CD2/CD48 interaction relies on equivalent, weak enthalpic and entropic effects, revealing a degree of plasticity in the interactions of CD2 with its ligands in the course of their evolution [30]. The interaction of CD2 with CD58 is rigid‐body in character (Figure 2f). A model of the intact, glycosylated form of the human CD2 ECD is shown in Figure 2g.^1^

## 3 CD45

**FIGURE 3:**
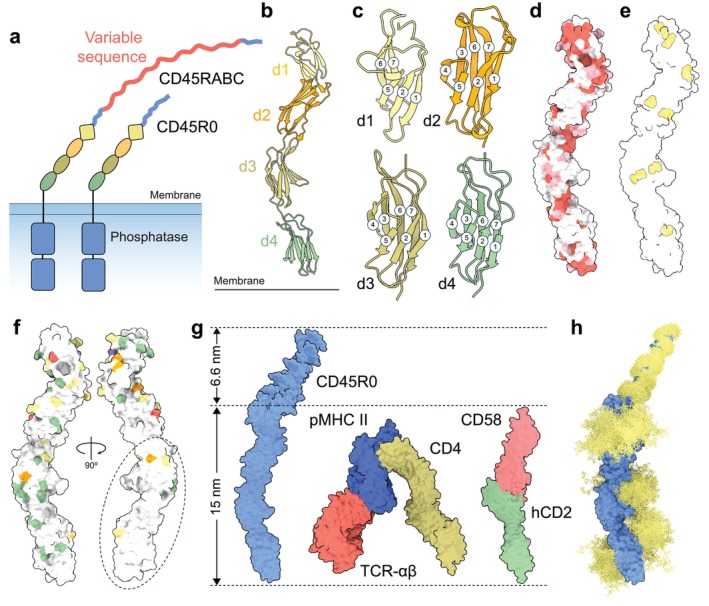
CD45. (a) Organization of the CD45 isoforms, CD45RABC and CD45R0. The extra mucin‐like protein sequence generated by alternative splicing of exons 4, 5, and 6 of the *PTPRC* gene is indicated as a red insert in the ECD of CD45RABC. The folded and mucin‐like regions are drawn approximately to scale. (b) Crystal structure of d1‐d4 of the CD45 ECD (PDB: 5fmv) with each domain color‐coded as in panel a. (c) Ribbon diagrams of ECD domains d1‐d4, with β‐strands numbered sequentially; β‐strands 3 and 4 are missing in d1 and β‐strand 3 is truncated in d2. (d) Level of conservation of CD45 surface residues; residues present or conserved in 1–6 of 11 sequences are colored white, 7–8 pink, and 9–11 red. (e) Locations of disulfide bonds (represented as cystines) in the folded part of the CD45 ECD. (f) Locations of glycosylation sequons for seven mammalian species mapped onto the CD45 d1‐d4 structure. Sites occupied in one species are colored green, 2–3 yellow, 4–5 orange, 6 red, and in 7 species, purple. The oval marks the region mostly lacking glycosylation sequons. (g) Dimensions of the CD45R0 ECD measured using negative‐staining EM, versus those of the CD4/TCR/pMHC class II complex (PDB: 3t0e) and the modeled hCD2/CD58 complex. O‐linked glycans have been modeled onto CD45R0. (h) A model of the glycosylated CD45 ECD. N‐glycosylation (yellow), at sites observed to be occupied in the crystal structure of the d1‐d4 region of the CD45 ECD, was modeled using GlycoSHIELD (as in Figure 2g). Core 2 O‐linked glycans were positioned at serine and threonine residues in a model of the mucin‐like region of CD45R0 based on the negative‐staining EM analysis, also using GlycoSHIELD.

The RPTP CD45 is among the most highly expressed proteins on the surfaces of T cells (i.e., > 100,000 molecules per cell and > 3 molecules per TCR) [20]. Since its catalytic efficiency is also very high (10‐ to 1000‐fold greater than that of relevant kinases [70]), CD45 is very likely to constrain tyrosine phosphorylation in resting cells. But CD45 is also needed to initiate signaling, in part owing to its activating effects on Src kinases [71, 72]. Expression of the multiple isoforms of CD45, whose ECDs consist of shared folded regions and variable mucin‐like segments, changes with cell type, developmental stage, and cell‐activation state [73]. The distinct isoforms result from alternative splicing of exon 4‐, 5‐, and 6‐encoded regions of *PTPRC* transcripts, with each region encoding, in turn, extra mucin‐like protein sequence. The ECD of CD45R0 has none of this extra sequence and consists of the shared folded region and a mucin‐like segment of 41 residues only, whereas the longest isoform, CD45RABC, has all the extra sequence and a mucin‐like segment of 202 residues (Figure 3a).

The crystal structure of the folded region of human CD45, solved to 2.9 Å resolution [31], showed that it comprises a ‘beads on a string’ array of four concatenated modular domains (Figure 3b). Despite being fibronectin domain 3 (FN3)‐related, d1 and d2 exhibit striking levels of degeneracy insofar as they lack or have unique arrangements of certain β‐strands (Figure 3c). According to sequence comparisons, the d1d2 region of CD45 has exhibited considerable structural plasticity in the course of vertebrate evolution [31]. Overall, the level of sequence identity shared by CD45 ECDs from mammals is extremely low (< 15%), contrasting with, e.g., the ECDs of type II RPTPs (> 90% identity) [74]. What conservation there is maps mostly to the domain interfaces (Figure 3d). The domains are connected by unusually short linkers versus other large RPTPs [74, 75], ensuring that there is extensive inter‐domain contact and that, overall, the assembly comprises a single rigid unit as confirmed by the largely identical copies of the folded d1‐d4 region in the asymmetric unit [31]. Similarly, whereas FN3 domains typically lack cysteines, the d1‐d4 region is 'cross‐linked' by eight disulfide bonds (Figure 3e). Overall, most glycosylation sites of d1‐d4 are poorly conserved across mammals, and the d1d2 region is likely to be more heavily glycosylated than d3d4. Intriguingly, one side of the d3d4 region is largely free of putative glycosylation sites (Figure 3f). This may help CD45 to preferentially access target proteins with relatively small ECDs, such as the TCR. On the other hand, if their cytosolic regions are unstructured and extended, close access of CD45 to its substrates might not be important. Consistent with the free diffusion of CD45 monomers at the cell surface [76], the CD45 ECD did not form dimers in the crystal lattice. We were also unable to identify a conserved candidate ligand‐binding surface [31].

By fusing the full‐length ECD of CD45R0 to the globular ∼70 kD ECD of a semaphorin [77], the length and flexibility of the mucin‐like segment of CD45R0 could be determined using negative staining electron microscopy [31]. This region of CD45R0 proved to be 6.6 nm in length (Figure 3g) and, since a full reconstruction was possible [31], it must also be rigid, as expected for mucins of this length [78]. Variable‐angle TIRF‐based imaging experiments implied that the ECDs of CD45R0 and CD45RABC are ‘upright’ at the cell surface [31]. A model of the intact, glycosylated form of the CD45 ECD is shown in Figure 3h.

## 4 The pMHC‐Bound αβ TCR

The ɑβ TCR consists of four non‐covalently associated, disulfide‐linked dimers, TCR‐ɑβ, CD3‐εδ, CD3‐εγ, and CD3‐ζ_2_. TCR‐ɑβ subunits are responsible for ligand reactivity via their variable regions and do not directly drive signaling. Instead, phosphorylation of the cytosolic regions of the three covalent CD3 dimers initiates intracellular signaling [79]. Prior to the structures of fully‐assembled TCR complexes becoming attainable, nuclear magnetic resonance (NMR)‐based and crystal structures of the subunits, mostly expressed in soluble forms, were determined [80, 81, 82, 83, 84, 85]. These pioneering studies established that TCR/pMHC recognition occurs in a conserved manner, but with no common binding mode explaining MHC restriction [86]. In addition, for the most part, the very small differences in the structures of ligated and unligated TCR‐ɑβ ECDs seemed to indicate that these parts of the receptor do not undergo shared conformational rearrangements that might otherwise have explained how TCR ligands differing in quality produce distinct signaling outcomes [86].

Discerning whether or how the intact TCR responds to ligand required structures of fully assembled ligated and unligated TCRs to be determined. Using cryo‐electron microscopy (cryo‐EM)‐based analysis of detergent‐solubilized receptors, Dong et al. reported the first structure of an intact ɑβ TCR, solved to 3.7 Å global resolution, in 2019 [87]. This confirmed that the receptor has TCRαβ/CD3δγε_2_ζ_2_ stoichiometry and offered two crucial insights into receptor assembly unobtainable from the earlier structural studies. First, assembly depends on interactions between the transmembrane (TM) regions of the TCR‐ɑβ heterodimer and CD3 dimers, forming a relatively tight eight‐helix bundle [87]. The TCR‐ɑ and TCR‐β TM regions associate via hydrophobic contacts and interact with each of the CD3 dimers via electrostatic contacts. Second, the new work revealed the nature of the interactions between the ECDs of the CD3‐εδ, CD3‐εγ, and TCR‐ɑβ heterodimers, which help to stabilize the extracellular parts of the receptor in an apparently rigid arrangement alongside interactions involving the membrane‐proximal connecting peptides (CPs) of each ECD pair, producing a “trimer‐like” arrangement of the receptor subunit ECDs [87]. No density was observed for the cytosolic regions of any receptor chain, implying that they are largely unstructured [87]. Subsequent refinement of the model to 3.2 Å resolution by the same team resulted in the identification of cholesterol moieties at two sites in the TM assembly [88]. Mutation of the cholesterol‐binding residues in the TM region led to TCR hyper‐reactivity, along with a 7.7 Å shift in the position of one of the CD3‐ζ chains [88]. Possible roles of cholesterol in αβ‐ and γδ‐TCR signaling are considered below.

**FIGURE 4:**
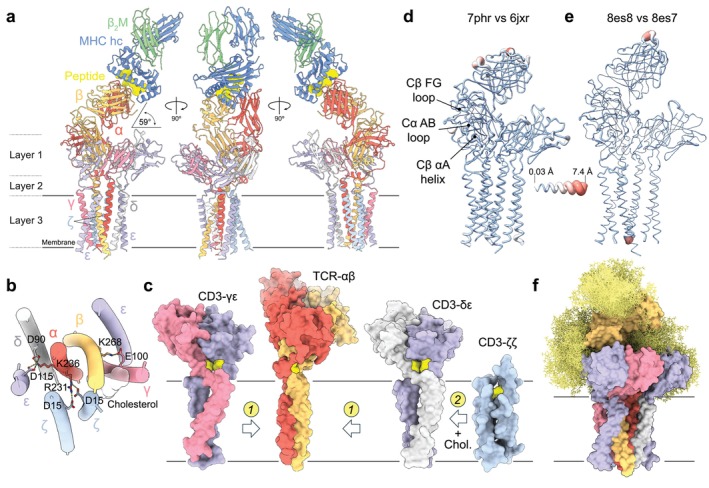
The αβ TCR. (a) Model of the fully‐assembled gp100/HLA‐A2/GPa3b17 TCR complex (PDB: 7phr) viewed along the plane of the membrane. Protein subunits are shown in ribbon format and the gp100 peptide antigen as a space‐filling model; the approximate boundaries of the membrane are shown as gray lines. The 59° tilt in the position of the complexed pMHC/TCR ECDs versus the plane of the membrane, and the three structural layers underpinning the assembly of the TCR complex are indicated. (b) Arrangement of the Layer 3 TM regions viewed toward the cell along an axis orthogonal to the membrane. Residues forming salt bridges are indicated. (c) Likely assembly intermediates in complex formation. The Layer 2 linker regions of the TCR‐αβ and CD3‐δε and ‐γε heterodimers are each stabilized by disulfide bonds (yellow). The 'tilting back' of the ECDs of the TCR‐αβ and CD3‐δε and ‐γε subunits might, especially, facilitate the close apposition of their TM regions, allowing charged TM‐residue interactions during step 1 of assembly. Cholesterol and CD3‐ζζ homodimer recruitment would complete assembly in step 2. (d, e) Structural differences between the gp100/HLA‐A2/GPa3b17 ligated TCR complex and the apo form of the TCR solved by Dong et al. [87] (PDB: 6jxr) (d), and the ligated (PDB: 8es8) and apo (PDB: 8es7) forms of the PN45545 TCR solved by Saotome et al. [90] (e), are shown (the representation is the same as in Figure 2f, except that the ligated structures are used for the putty diagrams). (f) Modeling of glycosylation (yellow) at sites observed to be occupied in the cryo EM structure of the GPa3b17 TCR, using GlycoSHIELD (as in Figure 2g).

These seminal studies offered the first insights into the structure of the resting state of the fully assembled TCR. To isolate a ligand‐bound receptor, we used a clonotypic, affinity‐matured TCR. Our expectation was that the best‐folded receptors would be those that reach the cell surface and that, if they had sufficient affinity, these TCRs could be ‘tagged’ prior to cell lysis with soluble pMHC monomers carrying a purification tag. The αβ TCR we assembled, called GPa3b17, binds with an affinity (*K*_D_) of 13 pM to a tumor antigen, that is, a glycoprotein 100‐derived peptide, gp100, complexed with HLA‐A2 [89]. The gp100/HLA‐A2/TCR complex, solubilized in the detergent glyco‐diosgenin and comprising 11 individual proteins and peptides (Figure 4a), was solved to a global resolution of 3.08 Å. This first view of pMHC recognition outside a crystal lattice confirmed that it occurs in the complementarity determining region (CDR)‐dependent, ‘diagonal’ binding mode observed in the crystals [86]. The improved binding of the affinity‐matured TCR to gp100/HLA‐A2 was explained by direct and indirect effects of CDR substitutions on both HLA‐A2 and gp100 recognition. The TCR‐αβ subunits, bound to gp100/HLA‐A2, are cradled by the ECDs of the CD3‐εδ and CD3‐εγ heterodimers, supported by the eight‐helix TM bundle (Figure 4a). Interactions with the CD3 subunits position the pMHC binding site of the TCR toward the ‘side’ of the complex and lock the TCR‐αβ subunit/pMHC complex at an angle of 59° relative to the plane of the membrane (Figure 4a). These interactions comprise multivalent contacts across two structural layers involving the CD3‐εδ and CD3‐εγ ECDs and the constant (C) regions and CPs of TCR‐αβ, an additional layer of contacts involving the TCR‐αβ TM bundle that crucially allows charged TM‐residue neutralization (Figure 4b), and interactions with cholesterol moieties incorporated into the TM bundle (Figure 4b) [32].

We were also interested in how such an assembly might form, having observed that ligand‐binding complexes lacking CD3‐ζ can be expressed at high levels in Chinese hamster ovary (CHO) cells [32]. We noticed that the CPs of the TCR‐αβ and CD3 heterodimers create distinctive, rigid links to their TM regions (Figure 4c). A short helix from TCR‐α fills the space between TCR‐Cβ and the N‐terminal ends of the TCR‐αβ TM helices, an arrangement reinforced by a disulfide bond to the TCR‐β CP. The linkers of each CD3 heterodimer, on the other hand, are stabilized by short β‐strands and paired disulfide bonds formed by vicinal cysteines in conserved CXXC motifs next to their TM regions, adjacent to and above the TCR‐αβ TM bundle. The hydrophobic interaction of cystines in this manner seemed to be unprecedented. The CPs, which also impose tilts in the CD3 heterodimer ECDs relative to their long axes (Figure 4c), may in this way facilitate the rigid‐body, close packing of the TM regions of the three heterodimers, in an arrangement stabilized initially by TM‐region salt‐bridges and then by interactions of their ECDs, explaining the formation of the CD3‐ζζ homodimer‐free complexes we detected in CHO cells. In a straightforward way, docking sites for cholesterol and CD3‐ζζ homodimers would be created, allowing completion of assembly.

Most importantly, our work allowed a first analysis of the effects of pMHC binding on the fully assembled TCR. Comparisons of the backbone conformations of our pMHC‐bound GPa3b17 TCR and the unligated structure solved by Dong et al. [87] indicated that the TCR resists ligand‐induced changes: the root‐mean‐square‐deviation between the two structures for all Cα atoms was 1.2 Å (Figure 4d) [32]. Previously, the binding of pMHC by the TCR was proposed to induce structural changes in four regions: (1) the TCR‐Cβ FG loop, (2) the TCR‐Cα AB loop, (3) the TCR‐Cβ αA helix, and (4) the TM regions, but these regions were essentially unchanged [32]. Only very minor displacements were observed at the C termini of the TM regions of CD3‐ε and CD3‐ε' (of 2.5–3.5 Å), perhaps only reflecting differences in construct design [32]. A caveat of these observations was that the strong binding of the GPa3b17 TCR could prevent the complex from accessing dynamic states used by more physiological, lower‐affinity TCRs during signaling. However, molecular dynamic simulations suggested that lower‐affinity TCRs would behave similarly, as was quickly confirmed by Saotome et al. [90], who determined unbound and ligand‐bound structures of a native, low affinity (*K*_D_ = 6 μM) TCR (PN45545; Figure 4e). A model of the intact, glycosylated form of the GPa3b17 TCR in the absence of ligand (Figure 4f) suggests that surprisingly large areas of the surface of the receptor will be occluded by N‐linked glycans, likely preventing receptor oligomerization.

## 5 The γδ TCR

γδ T‐cells comprise a T‐cell lineage that is distinct from αβ T‐cells and contributes to tumor and mucosal immunity [91]. In contrast to the αβ TCR, the γδ TCR reacts with a variety of structurally diverse ligands. These include the stress‐induced MHC I‐like molecules, CD1 and MR1, and non‐MHC‐like ligands, such as butyrophilin and butyrophilin‐related proteins [92]. From the outset, structural work on the γδ TCR suggested that γδ and αβ TCRs would be substantially different. In the TCR‐γδ ECD structure solved by Allison et al. [93], the C domains “swung out” from underneath the V domains versus the arrangement seen in TCR‐αβ ECDs. In addition, no conserved surfaces in the Cα/Cβ and Cγ/Cδ domains seemed likely to mediate shared CD3 subunit interactions. The TCR‐γ chain also lacked the extended FG loop of TCR‐β that has been linked to mechanotransduction [94], and Cδ had conventional Ig superfamily domain topology with C, F, and G β‐strands, in contrast to Cα whose topology is unique. Finally, Allison et al. [93] noted, like others previously [95, 96], that the TCR‐γ and ‐δ subunits have substantially longer CPs.

**FIGURE 5:**
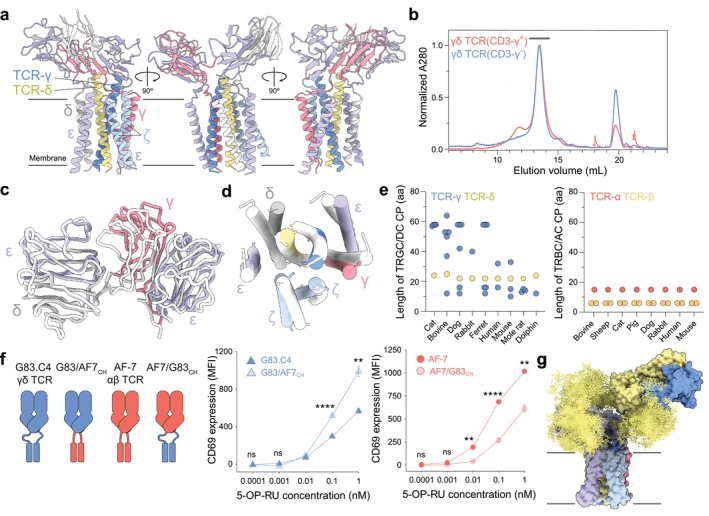
The γδ TCR. (a) Model of the fully‐assembled MR1‐specific G83.C4 γδ TCR (PDB: 9cia) viewed along the plane of the membrane. Protein subunits are shown in ribbon format; the approximate boundaries of the membrane are shown as gray lines. (b) Elution of the fully‐assembled G83.C4 γδ TCR, and a form of the complex expressed in the absence of the CD3‐γ chain, from an analytical Superose 6 Increase 10/300GL size‐exclusion chromatography column. The horizontal bar indicates the elution position of material used for structural analysis of the fully‐constituted complex. (c) Displacement of the CD3 ECDs within the γδ‐ (colored) and αβ‐ (PDB: 7phr; white) TCR complexes, observed after superimposing the CD3‐δε heterodimer ECDs. The centers of mass of the CD3 subunit ECDs differ by up to ~8 Å between the complexes [33]. (d) Conservation of organization of the TM regions in the γδ TCR (colored) versus the apo αβ TCR (PDB: 7phr; white). (e) Differences in TCR CP sequence lengths for γδ and αβ TCRs from different mammals. (f) Effects of αβ and γδ TCR flexibility on signaling outcomes. A schematic of the G83.C4 and AF‐7 wild‐type and chimeric (CH) TCR constructs wherein, for the chimeras, the variable and constant domains were interchanged between the two receptors, is shown on the *left*. Mean fluorescence intensities (MFI) for CD69 expression are also shown, for Jurkat T‐cells expressing G83.C4 and G83/AF7_CH_ TCRs (*middle*), and AF‐7 and AF7/G83_CH_ TCRs (*right*), following stimulation with C1R cells treated with different amounts of 5‐OP‐RU. Reducing the flexibility of the γδ TCR increased signaling whereas enhancing it for the αβ TCR reduced signaling. *p* values are indicated: **< 0.01; ****< 0.0001. Error bars represent standard deviations. Statistical comparisons were performed using one way analysis of variance with Tukey's multiple comparison test; two independent experiments with *n* = 3 cocultures each were analyzed. (g) Modeling of glycosylation (yellow) at sites observed to be occupied in subunits of the γδ TCR (PDB: 9cia), using GlycoSHIELD (as in Figure 2g).

Two structures of detergent‐solubilized, fully assembled γδ TCRs solved using cryo‐EM were reported by Xin et al. [97], followed soon after by a third structure determined by us (Figure 5a) [33]. Additional work by Saotome and colleagues [98] on the γδ TCR clonotypes studied by Xin et al. extended and confirmed all of the initial findings. The TCRs studied by Xin et al. were of the Vγ9Vδ2 and Vγ5Vδ1 subtypes, whereas we solved the structure of a Vγ8Vδ3 TCR whose interactions with MR1 are well‐characterized [99, 100]. We reasoned that although MR1‐reactive T cells comprise only a minor subset of circulating γδ T‐cells [99, 100], because the C regions of γδ TCRs seemed likely to determine their overall organization, whatever was learned about the Vγ8Vδ3 TCR would probably apply to receptors comprising other combinations of Vγ and Vδ domains. The study of an MR1‐binding γδ TCR would also allow direct functional comparisons with MR1‐binding αβ TCRs. Whereas γδ TCR stoichiometry had been controversial [101, 102], the solved structures all consisted of 1:1:1:1 associations of TCR‐γδ heterodimers with CD3‐δε, ‐γε, and ‐ζζ dimers (Figure 5a), although a γδ TCR lacking CD3‐γ could also be purified (Figure 5b). Once again, very little, if any, density was observed for the cytosolic regions of the TCR‐γδ and CD3 subunits, confirming their flexibility.

The first key observation was that the overall organization of the CD3 ECDs in both TCR subclasses is largely conserved [33, 97], despite the limited number of pairwise interactions stabilizing the positions of the CD3εδ and CD3εγ heterodimers (Figure 5c) [33]. Residues contributing to a three‐way interaction that position CD3‐εγ next to TCR‐β and CD3‐εδ alongside TCR‐α were poorly resolved in the γδ TCR [33], perhaps because stabilizing contacts with the TCR‐γδ C domains are absent. Instead, the positioning of the CD3‐εδ and CD3‐εγ heterodimer ECDs relies on the relatively rigid linker regions packing the CD3 ECDs against the top of the TM helix bundle. For the γδ and αβ TCRs, the centers of mass of the CD3‐δε and ‐γε ECDs differ by as much as 8 Å [33], suggesting that precise positioning of the signaling subunit ECDs of TCRs is unimportant.

The second important finding was that the organization of the TM regions of γδ and αβ TCRs was very similar, despite the limited similarity of the TCR‐γ/TCR‐δ and TCR‐α/TCR‐β TM region sequences (Figure 5d) [33, 97]. Whereas, for the αβ TCR, TM region packing depends partly on interactions in the membrane‐proximal regions of the subunit ECDs [32], the γδ TCR relies almost exclusively on the electrostatic interactions of conserved, charged residues in its TM region. Cholesterol is consistently incorporated into the TM regions of γδ TCRs as in the case of ɑβ TCRs, but the distribution appears distinct (Table 1). Whereas robust density is seen in the outer leaflet‐embedded region of the ɑβ TCR, similar‐quality density is only seen in the inner leaflet‐associated portion of the γδ TCR [33, 97]. The outer leaflet site in the γδ TCR is blocked by the reorientation of a CD3‐ζ residue (Lys30) toward a TCR‐γ TM region residue (Met254) [33]. Mutations that reduced cholesterol binding lowered signaling by the γδ TCR [97], but the changes in cholesterol levels and their effects on signaling were each relatively modest. Cholesterol de‐sequestration did, however, initiate spontaneous T‐cell activation [97]. But if cholesterol stabilizes the assembly, its removal could induce receptor aggregation and signaling. Considering the native structures only, given (1) that the overall organization of the TM domains is largely unaffected by the presence of cholesterol in the inner versus the outer leaflet TM regions, and (2) that the sterol is present in both ligated and unligated TCRs, it seems more likely that cholesterol affects receptor assembly rather than signaling.

**TABLE 1:** Summary of locations of cholesterol in solved αβ and γδ TCR structures.

PDB ID	7fjd	8es7	8es8	8es9	7phr	8jc0	9cia
TCR	αβ	αβ	αβ	αβ	αβ	γδ	γδ
Apo	×	×				×	×
Ligated			×	×	×		
Cholesterol (outer leaflet)	×	×	×	×	×		
Cholesterol (inner leaflet)	×					×	×

Easily the most striking and telling difference between the two subclasses of receptors, however, was that, in contrast to the αβ TCRs [32, 87, 90], for all three γδ receptors studied, the TCR‐γδ ECDs were either absent from the density or the associated density was discontinuous with that of the CD3 assembly [33, 97]. The mobility of the γδ TCR is explained by several differences between the two receptor subclasses. First, αβ and γδ C domain sequences are poorly conserved (~12% and 25% identity for C‐δ/C‐α and C‐γ/C‐β comparisons, respectively), and the absence, in the FG loop of TCR‐γ and the DE loop of TCR‐δ, of residues forming important electrostatic contacts prevents the TCR‐γδ heterodimer from associating with the CD3 ECDs in the same way [33]. Automated structure comparisons ranked numerous antibody C domains ahead of C‐β and C‐α domains as the structures most like C‐γ and C‐δ, respectively. Second, as noted [93, 95, 96], the lengths of the membrane‐proximal C‐γ and C‐δ CPs are significantly longer (by 14 and 13 residues, respectively, depending on exon usage) than the equivalent CPs of the αβ TCR, and the CPs are unstructured or unresolved in the cryo‐EM maps. TCR‐γ CPs are typically encoded by multiple exons, vary significantly in length among mammals, and can be as long as sixty residues (Figure 5e). The length of the single TCR‐δ CP is, however, highly conserved between species and constrained to 22–25 residues only, limiting the maximum 'reach' of the receptor [33].

We directly tested the impact of TCR flexibility on its function [33]. The αβ TCR, AF‐7, and the γδ TCR, G83.C4, bind with similar affinity and in a similar head‐to‐head orientation to a shared ligand, MR1, presenting the bacterial metabolite 5‐(2‐oxopropylideneamino)‐6‐D‐ribitylaminouracil (5‐OP‐RU) [100]. T cells transduced with the AF‐7 ɑβ TCR signaled more potently in response to MR1/5‐OP‐RU than cells bearing the G83.C4 γδ TCR [33]. Importantly, when the variable regions of the G83.C4 γδ TCR were grafted onto the presumably rigid AF‐7 TCR scaffold, T cells expressing the chimeric receptor produced much stronger signaling responses, measured as CD3ζ phosphorylation, which were comparable to those produced by AF‐7, suggesting that the flexibility of the γδ TCR constrains its responsiveness [33]. We also find that reciprocal swaps of all of the V and C domains between the two receptors increase signaling following G83.C4‐based MR1/5‐OP‐RU recognition (i.e., after ‘rigidifying’ the G83.C4 TCR), and reduce it after AF‐7–dependent ligand binding (i.e., when the AF‐7 TCR becomes more flexible; Figure 5f). Xin et al. found that T cells expressing a γδ TCR comprised of the Cγ region encoded by *TRGC1* produced stronger responses to the MHC‐like ligand, CD1d, than cells expressing receptors with the 16‐residue longer CPs encoded by *TRGC2* [97], also suggesting that the flexibility of the γδ TCR limits its reactivity. We proposed that the reduced reactivity of the more flexible γδ TCR reflects a compromise between it having to bind structurally diverse ligands, and having to engage them efficiently. A model of the intact, glycosylated form of the G83.C4 γδ TCR is shown in Figure 5g.

Xin et al. were also able to characterize the structure of the flexible ECD of a Vγ5Vδ1 TCR [97] because, unexpectedly, this TCR, but not the Vγ9Vδ2 or Vγ8Vδ3 TCRs [33, 97], formed receptor dimers. Dimer formation relied on homotypic interactions involving residues in the invariant HV4 segment of Vγ5 that differ in Vγ9 or Vγ8 domains, with other contributions from CDR2; these residues are also present in Vγ2 domains, which also dimerized in solution [97]. Mutating these residues prevented dimerization of purified Vγ5Vδ1 receptors and impaired ligand binding and T‐cell signaling, suggesting that dimerization potentiates signaling by increasing ligand binding avidity. Similar observations were made by Hoque et al. in their studies of the same receptor [98].

## 6 Accessory Receptors: CD28, CTLA‐4, and PD‐1

The covalent homodimers, CD28 and CTLA‐4, are strong modulators of T‐cell responses. Their similar architectures comprise ECDs with single V‐set Ig superfamily domains and stalks linked to TM and cytosolic regions. The receptors bind via “MYPPPY” motifs [103] to two shared ligands: a weak dimer, CD80, and the monomer, CD86 [103, 104, 105]. CD28 and CTLA‐4 have orthogonal functions: CD28 provides signals essential for full T‐cell activation, whereas CTLA‐4 suppresses T‐cell responses [103, 106]. The binding affinities and valencies of CD28 and CTLA‐4 also differ substantially [107]. PD‐1 (programmed cell death protein 1) is among the most potent inhibitory receptors in the immune system, and the pre‐eminent target of transformative immunotherapies [108]. PD‐1 consists of a single extracellular V‐set Ig superfamily domain also attached via a stalk to TM and cytosolic regions. The TM region is proposed to dimerize PD‐1 at the cell surface [109]. Its two ligands, PD‐L1 and PD‐L2, differ 3–4 fold in affinity for the receptor [38]. We helped determine the structure of the CTLA‐4/CD80 ECD complex [35] and published the structure of the apo CTLA‐4 ECD [36] in 2001 and 2011, respectively. Our structure of a human CD28 ECD monomer, complexed with the fragment antigen‐binding (Fab) of a mitogenic antibody, 5.11A1, was published in 2005 [34]. Finally, our collaborators' structures of the apo PD‐1 ECD [38] and of the PD‐1 ECD complexed with our antibody agonist, Clone 19 [110], were reported in 2013 and 2024.

### 6.1 CD28

**FIGURE 6:**
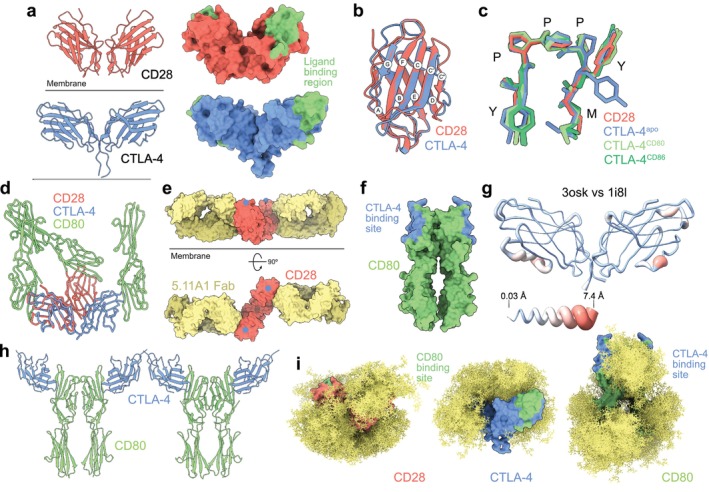
CD28 and CTLA‐4. (a) Ribbon and surface‐rendered views of the ECDs of apo CD28 (PDB: 1yjd) and CTLA‐4 (PDB: 3osk). The ligand (i.e., CD80) binding surface of CTLA‐4 incorporating the MYPPPY sequence, and the equivalent region of CD28, are indicated. (b, c) Superimposition of the apo CD28 and CTLA‐4 (chain A) Ig superfamily domains (b), and of the FG loop MYPPPY sequences of CD28 and the apo and complexed forms of CTLA‐4 (with CD80, PDB: 1i8l; with CD86, PDB: 1i85) (c). (d) The d2 clash resulting from superimposing CD80 monomers with the CD28 homodimer in the manner of CD80 binding by CTLA‐4 (PDB: 1i8l); for the CTLA‐4 homodimer, which forms a complex in which the CD80 monomers are parallel, there is no such clash. (e) Orthogonal views of the crystallographic CD28 ECD homodimer/5.11A1 Fab complex. The view in the upper panel is along the plane of the membrane. The position of a residue equivalent to one in rat CD28, which disrupts the binding of a non‐mitogenic anti‐rat CD28 antibody when mutated [112], is marked with a blue circle. (f) The homodimer formed in crystals of the CD80 ECD (PDB: 1dr9); the location of the CTLA‐4 binding site, determined subsequently [35], is indicated. (g) Structural differences between the CD80‐ligated (PDB: 1i8l) and apo CTLA‐4 homodimer (PDB: 3osk) (see Figure 2f for details of the representation). (h) The repeating array of CTLA‐4 and CD80 ECD homodimers observed in crystals of the complex (PDB: 1i8l). (i) Modeling of glycosylation (yellow) at sites found to be occupied in crystals of CD28 (PDB: 1yjd; predicted CD80 binding site, green), CTLA‐4 (PDB: 3osk; CD80 binding site, green), and CD80 (PDB: 1dr9; CTLA‐4 binding site, blue), using GlycoSHIELD (as in Figure 2g).

CD28 enhances T‐cell activation via the PI3‐kinase pathway; in the absence of CD28 engagement, TCR signaling drives T cells into an anergic state [111]. Following unsuccessful attempts to crystallize intact, deglycosylated CD28 ECD homodimers, the structure of reduced, glycosylated monomers bound to the 5.11A1 Fab [112] was determined instead [34]. A major lattice contact in the crystals produced a plausible model of the CD28 ECD homodimer (Figure 6a, upper) similar to the known CTLA‐4 dimer (Figure 6a, lower). However, whereas CTLA‐4 homodimerization relies on residues next to and including the stalk‐like region, the CD28 lattice contact was formed by a small, three‐stranded A′G′F β‐sheet present in V‐set Ig superfamily domains [34]. Single‐particle cryo‐EM analysis of an intact CD28‐Fc fusion protein bound to the 5.11A1 antibody showed that the lattice‐based CD28 dimer was a better fit for the electron density than a CTLA‐4–based model. Moreover, mutations of residues mediating the lattice contact prevented CD28 expression at the cell surface, whereas mutations of residues equivalent to those involved in CTLA‐4 dimerization had smaller effects [34]. Our lattice‐based homodimer is therefore very likely to mimic the native CD28 ECD.

The V‐set Ig superfamily domain of CD28 is structurally most similar to that of CTLA‐4 [34]. Variations are found in the G β‐strand of CD28, as well as a reduction in the length of the CD28 CC' loop versus that of CTLA‐4, which would have prevented CD28 dimerization in the way observed in the crystal (Figure 6b). The structure of the shared ligand‐binding MYPPPY motif of CD28, with the *cis‐trans‐cis* conformations of the three proline residues, is virtually identical to that of CTLA‐4 (Figure 6c). The shared surface used by CTLA‐4 to bind to both CD80 and CD86 is preserved in CD28, but the surfaces mediating CTLA‐4/ligand complex formation outside this region differ in CD28 and account for the reduced affinity of CD28 for its ligands [34, 107]. The lattice‐based CD28 dimer slightly reorients each subunit, explaining the differences in the valency of CTLA‐4 and CD28 [34]. Docking experiments suggested that bivalent ligand‐binding by CD28 is prevented by a steric ‘clash’ in the membrane‐proximal domains of bivalently‐bound CD80 (Figure 6d), and that CD86 binding will be greatly constrained in the same manner. The mitogenic 5.11A1 antibody bound CD28 bivalently via a membrane‐proximal epitope, along an axis parallel to the membrane (Figure 6e), in contrast to non‐mitogenic antibodies that bind epitopes further away from the membrane [34, 112]. This suggested to us that the mitogenic antibody could induce signaling by locally excluding RPTPs such as CD45, when the antibody also forms complexes with Fc receptor‐expressing cells [34], as we subsequently demonstrated [110]. Our structure of the ECD of the CD28 and CTLA‐4 ligand, CD80 (Figure 6f), published in 2000 [37], revealed that it forms a 2‐fold rotationally symmetric homodimer and consists of a novel combination of Ig superfamily domains, that is, a V‐set domain found in adhesion proteins and a C1‐set domain typical of antigen receptors.

### 6.2 CTLA‐4

CTLA‐4 suppresses signaling in T cells by recruiting cytosolic tyrosine phosphatases, for example, Src homology 2 domain‐containing tyrosine phosphatases (SHP) 1/2, which reverse tyrosine phosphorylation, and/or by impairing CD28 signaling directly by competing for their shared ligands [103], or indirectly via the capture and degradation of the ligands by trans‐endocytosis [113]. As expected, the CTLA‐4 V‐set Ig superfamily domain^2^ is most similar to that of CD28, and to a lesser extent, PD‐1, rather than those of antigen receptors and adhesion molecules [35, 36, 114]. Dimerization relies on (1) intermolecular disulfide bond formation, (2) hydrophobic interactions of the Ig superfamily domains involving the A′ and G strands of each monomer, and (3) three networks of water molecules that help rigidify the stalk, perhaps favoring ligand engagement [35, 36, 114].

The MYPPPY segment of CTLA‐4 dominates interactions with its ligands [35, 114]. CTLA‐4 forms hydrophobic contacts with the mostly nonpolar surface of CD80 [35]. In further contrast to CD2 [29], the binding surfaces of CTLA‐4 and CD80 exhibit a high degree of shape complementarity [35]. The *Sc* metric quantifying this parameter [115] is 0.74–0.77 for the CTLA‐4/CD80 binding interface, which is considerably higher than that for CD2 and CD58 (0.58) [60], and even higher than that of antibody/protein antigen interfaces (0.64–0.68) [115]. However, the CD80 binding site of CTLA‐4 is relatively small, with only 460 Å^2^ of surface being buried per monomer [35] (for CD2 and CD58, 570–590 Å^2^ of surface is buried [60]). This helps to explain the fast kinetics of CTLA‐4/ligand interactions [107, 116].

The binding of CD80 to CTLA‐4 is accompanied by minimal changes in the CTLA‐4 ECD (Figure 6g) [36]. Notably, the Ig superfamily domains of the apo ECD homodimer, which are very similar, both exhibit more similarity with equivalent domains in the ligated structures than they do to each other, emphasizing the rigid‐body nature of complex formation. There is a small rotation in the sidechain of M99 (part of the MYPPPY motif) when binding CD86, as well as some changes in the position of the C‐terminus of the G strand, neither of which occurs when CTLA‐4 binds CD80 [36]. There are also small shifts in the positions of the C″ strand and the BC and CC′ loops. Because these changes are not produced by both CD80 and CD86, they are unlikely to function allosterically. Despite binding the shared MYPPPY motif, CD80 and CD86 interact with CTLA‐4 in different ways [35, 36, 114]. Whereas minimal reorganization of CD80 is needed to accommodate CTLA‐4, CD86 undergoes induced‐fit including, e.g., the rotation of the FG loop allowing contacts to form with the MYPPPY motif. Otherwise, ligand binding is rigid‐body in character [36]. Strikingly, in crystals, the CTLA‐4 and CD80 ECD dimers form a zipper‐like arrangement (Figure 6h), revealing a structural basis for the formation of very stable inhibitory complexes at the T‐cell surface [35]. Models of the intact, glycosylated forms of the CD28, CTLA‐4, and CD80 homodimer ECDs are shown in Figure 6i.

### 6.3 PD‐1

**FIGURE 7:**
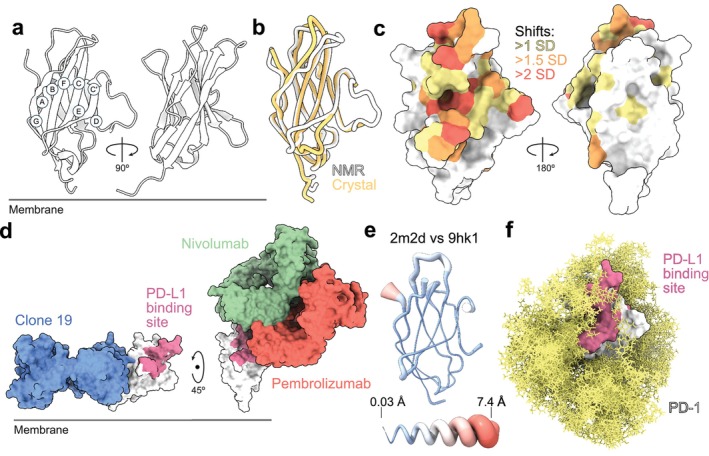
PD‐1. (a) Ribbon representation of the PD‐1 ECD (PDB: 2m2d). (b) Comparison of the NMR and the crystal (PDB: 3rrq) structures of the PD‐1 ECD. (c) Surface renderings of PD‐1 (PDB: 2m2d) showing how the structure is affected by ligand binding, based on how the backbone (^15^N, ^13^C′, and ^1^H^N^) NMR signals of surface residues are perturbed by PD‐L1. Residues with minimal shift values lower than 1 standard deviation (SD) versus the average for all residues are colored white, those > 1 SD yellow, > 1.5 SD orange, and residues with minimal shifts > 2 SD are colored red. Both the ligand‐binding face and the back of the molecule are shown. Very similar data were obtained for PD‐L2 binding [38]. (d) Crystal structures of the complexes of Clone 19 (PDB: 9hk1), nivolumab (PDB: 5wt9), and pembrolizumab (PDB: 5b8c) antibody Fv or Fab fragments, with the PD‐1 ECD. For reference, the PD‐L1 binding site on PD‐1 is also indicated [118]. (e) Structural differences between apo PD‐1 ECD (PDB: 2m2d), and the PD‐1 ECD in the Clone 19 Fab/PD‐1 ECD complex (PDB: 9hk1; see Figure 2f for details of the representation). (f) Modeling of glycosylation (yellow) at sites found to be occupied in structures of the PD‐1 ECD, using GlycoSHIELD (as in Figure 2g).

Inhibitory signaling by PD‐1 is at least partially explained by its recruitment of the tyrosine phosphatases, SHP1/2, after it's phosphorylated on cytosolic signaling motifs following ligand binding [117]. NMR analysis of the human PD‐1 ECD^3^ showed that it comprises a two‐layer β sandwich with the topology of Ig superfamily V‐set domains, i.e., GFCC′ and ABED sheets stabilized by a disulfide bond (Figure 7a) [38]. Structure comparisons showed that it is most similar to the V‐set domains of antigen receptors and CTLA‐4, as anticipated by sequence comparisons [36]. The first PD‐1/PD‐L1 ECD complex solved (PDB: 3bik) revealed an *in trans* binding mode reminiscent of the *in cis* interactions of antigen receptor variable domains [119], suggesting an evolutionary path from *in trans* interacting monomeric receptors to *in cis* heterodimers, or *vice versa*. Compared with typical V‐set domains, however, the edge of the GFCC′ sheet of PD‐1 is unusually flexible, and it completely lacks a C″ strand. The crystal structure of the human PD‐1 ECD (PDB: 3rrq) confirmed that the C'D loop is unstructured and that the CC′ and FG loops are highly flexible (Figure 7b). The ligand‐binding sites of PD‐1 were mapped via perturbations of PD‐1 backbone NMR signals in the presence of soluble PD‐L1 and PD‐L2 [38] (Figure 7c). For PD‐L1, the changes were localized to a patch of surface residues on the GFCC′ β‐sheet: i.e., β‐strand C and the CC′ loop, β‐strand C′, the C'D loop, and β‐strands E and F, along with the FG loop, β‐strand G, and the BC and FG loops, defining the PD‐L1 binding site (as confirmed subsequently [118]). Although PD‐L1 and PD‐L2 bound the same region of PD‐1, for PD‐L2 there were fewer contributions from its BC and FG loops. Importantly, there was little or no indication that ligand‐induced changes occurred beyond the region of direct contact [38].

The possibility that the anti‐CD28 antibody we crystallized with CD28 was agonistic because it bound a membrane‐proximal epitope [34] prompted the generation of an anti‐PD‐1 antibody agonist, Clone 19, for use as a potential suppressor of inflammatory processes [110]. The structure of the ECD of PD‐1 complexed with the Fab fragment of Clone 19 (PDB: 9hk1) confirmed that the antibody binds to the base of β‐strands A, F, and G, on the opposite side of the protein from the PD‐L1/PD‐L2 binding site (Figure 7d) [110]. In contrast, ligand‐blocking anti‐PD‐1 antibodies used in the clinic, i.e., nivolumab and pembrolizumab, bind closer to the top of PD‐1 (PDB: 5b8c, PD‐1/pembrolizumab Fab complex; PDB: 5wt9, PD‐1/nivolumab Fab complex; Figure 7d) [110]. We found a striking inverse correlation between epitope position relative to the membrane and the level of agonistic signaling elicited, insofar as the three antibodies were agonistic in the following manner: Clone 19 > pembrolizumab > nivolumab [110]. This supported the general idea that the closer an antibody binds to the membrane, the better able it is to create small gaps between the receptor‐expressing target cell and Fc receptor‐expressing cells that co‐engage the antibody, leading to the more efficient exclusion of large RPTPs such as CD45 and stronger signaling [110]. Comparison of the apo PD‐1 ECD and the PD‐1 ECD/Clone 19 Fab complex indicated that the structural core of PD‐1 is largely unaffected by antibody binding (Figure 7e) [110], indicating that, like the native PD‐1 ligands, signaling by Clone 19 is not dependent on large‐scale structural rearrangements. A model of the intact glycosylated form of the PD‐1 ECD is shown in Figure 7f.

## 7 Conclusions

Our structural work on CD2 and its ligands, CD45, αβ‐ and γδ‐TCRs, and the accessory proteins, CD28 and CTLA‐4 and their ligand CD80, and PD‐1, offers a number of insights into the early events underpinning antigen detection and signaling at close contacts.

Its linker region, which lengthens CD2 and positions its ligand‐binding site close to the top of the protein, and its flexibility [27, 28], are likely to favor head‐to‐head binding of CD2 to its ubiquitously expressed and relatively abundant ligands and facilitate close‐contact formation during the early stages of cell–cell contact. The head‐to‐head lattice contact seen in all our CD2 ECD crystals provided a model of the ligand complexes that CD2 forms, measuring ~15 nm along an axis orthogonal to the membrane [27, 28]. These complexes are likely to set the ‘height’ of the close contact, and it is now clear that a large number of receptors form complexes matching these dimensions [120], allowing them to function at close contacts depending on when and where their ligands are expressed. The structural plasticity inherent in the complexes CD2 forms with its related ligands suggests that the evolutionary diversification of these proteins was rapid and constrained only by binding needing to be weak and specific, and in the case of CD2, its topology and dimensions being maintained, which will have required only that its linker region is conserved. Overall, CD2 and CTLA‐4 provide what are likely to be extreme examples of low‐affinity, highly‐specific protein binding modes at the cell surface: one reliant on poor surface‐shape complementarity and electrostatic contact (i.e., CD2) [29], and the other on small, hydrophobic surfaces with considerable shape complementarity (i.e., CTLA‐4) [35, 114].

The structural work confirmed that the ECDs of all forms of CD45 extend beyond the 15 nm inter‐membrane gap created by CD2 and its ligands at close contacts [31]. The smallest isoform of CD45, i.e., CD45R0, is 21.6 nm, and the RABC isoform is larger, as expected [31]. Size differences as small as 5 nm are enough to drive unbound proteins from membrane interfaces created by smaller complexes of binding proteins [121]. This indicates that all forms of CD45 would be physically excluded from close contacts created by CD2/ligand interactions, as we observed in the case of Jurkat T‐cells (Figure 1b [23]), which mostly express the smaller forms of CD45 [122]. Altogether, the structural data indicated that the fine‐structure of the CD45 ECD is less important than its mechanical properties given, on the one hand, the degenerate structures of its constituent domains and poor sequence conservation beyond the domain interfaces, and the tight domain packing and disulfide bond‐based ‘cross‐linking’ of the ECD, on the other [31]. This can be explained by the ECD of CD45 functioning primarily as a physical lever, driving the phosphatase out of close contacts [31].

Whereas the ligand‐binding site of CD2 is at the top [27, 28], the binding surfaces of the αβ TCR [32], CD28 [34], CTLA‐4 [35, 114], and CD80 [37] are all toward or on the side of each structure. For the αβ TCR, we proposed that positioning the ligand‐binding site in this way, rather than at the top, serves two important purposes [32]. First, the TCR does not have to form head‐to‐head complexes in the short period of time during which close contacts are created by CD2 and its ligands, or after contact is made and only ‘sideways’ movement is possible. Once close contacts form, the tilted arrangement of the TCR‐αβ subunits and the location of the pMHC ligand‐binding site on the side of the TCR would result in the TCR and pMHC‐binding sites overlapping, facilitating productive scanning by the TCR for rare pMHC ligands via sideways interactions. Second, TCR/pMHC binding in this manner positions the pMHC for favorable interactions with co‐receptors, explaining why their recruitment follows TCR/pMHC engagement and initial signaling [10]. We proposed that TCRs engaging pMHC with reverse topology [123] may fail to initiate strong signaling because the co‐receptor binding site is inaccessible once stable close contacts form [32]. Similar arguments can be applied to CD28 and CTLA‐4. These observations suggest that receptor topology could have a large impact on the sequence of signaling events at close contacts. In marked contrast to these proteins, the ligand‐binding regions of γδ TCRs are topologically unconstrained [33, 97] so that these receptors can engage a variety of structurally‐distinct ligands [92]. Strikingly, the maximum dimensions of the γδ TCR are nevertheless constrained by the fixed‐length TCR‐δ CP regions. This could ensure that highly flexible γδ TCRs are trapped in the shallow, robustly CD45‐excluding close contacts created by CD2 and its ligands rather than deeper regions of contact stabilized by larger e.g., ICAM‐1/LFA‐1 complexes, allowing more‐productive signaling.

But what of receptor triggering per se? The signaling receptors we chose to study belong to the set of ~100 “immune receptors” characterized by (1) having mostly small ECDs (< 20 nm), (2) their binding to ligands anchored to the surfaces of other cells, (3) having unstructured cytosolic regions with multiple tyrosine residues and no intrinsic catalytic activity, and (4) being phosphorylated and dephosphorylated by membrane‐associated, extrinsic tyrosine kinases, e.g., Lck, and phosphatases, such as CD45, respectively [120]. To us and to others [8, 120], these similarities imply that immune receptors are triggered via a single, shared mechanism, that is, they are 'all‐in or none‐in'. A corollary of this proposition is that insights obtained for any one receptor will apply to the others, which we used to justify our initial focus on receptors that could be studied easily. The work on CTLA‐4 offered the first and, perhaps, clearest evidence that immune receptor triggering does not invariably depend on spontaneous conformational changes [36]. This seems also to be true of αβ TCR triggering [32] and antibody‐induced signaling [110], but as we have sought to emphasize throughout, rigid‐body binding is a shared feature of all the interactions we have studied. Whilst the role of forces in immune receptor signaling has not been explored very broadly, we note that the γδ TCR is not a mechanosensor [124], ruling this out as a general requirement of immune receptor triggering. The structural basis for CD45 exclusion from close contacts formed by the interaction of CD2 with its ligands now seems to be settled, however. It follows that the phosphorylation of any immune receptor would be potentiated by being trapped in close contacts by its ligands, just as slowing the diffusion of the αβ TCR at close contacts suffices to initiate strong signaling [125], satisfying the all‐in or none‐in requirement.

## 8 Afterword

Recently, a preprint published by Notti et al. [126] addressed the question of how the αβ TCR might be configured in a membrane‐like environment, rather than in detergent micelles. In their experiments, Notti et al. reconstituted detergent‐solubilized TCRs into lipid nanodiscs containing a mixture of polar lipids and cholesteryl hemisuccinate, and determined their structures using cryo‐EM. Rather than the largely identical conformations of unligated and ligated receptors observed by ourselves and others for detergent‐solubilized receptors [32, 87, 90], “closed and compacted” conformations of the unligated receptor were observed that were 3.5 nm shorter than the ligand‐bound complex. These striking findings confound the notions (1) that CD2/ligand binding and close‐contact formation favors pMHC engagement by the TCR by creating an overlap in the positions of their binding sites [32], and (2) that the TCR engages ligands in a rigid‐body manner [32, 90]. It will be important to carefully determine the extent to which the TCR occupies these various states at the T‐cell surface.

## Conflicts of Interest

The authors declare no conflicts of interest.

## Data Availability

The data that support the findings of this study are available from the corresponding author upon reasonable request.

## Endnotes

^1^
To emphasize the impact of O‐ and/or N‐glycosylation on their structures and interactions, we have generated structural models of the proteins we have studied, each with 50 representative glycan conformers at each of the glycosylation sites found to be occupied with sugar during the structure determinations. The glycan conformers were generated from 3 μs molecular dynamics simulations by GlycoSHIELD [61]. No attempt has been made to model the constraining effects of the cell surface at membrane‐proximal sites.

^2^
The structure of the complex of soluble forms of CD80 and CTLA‐4 was solved using the coordinates of the CD80 ECD structure [37], by Stamper et al. of Wyeth Research [35].

## References

[1] R. N. Germain , E. A. Robey , and M. D. Cahalan , “A Decade of Imaging Cellular Motility and Interaction Dynamics in the Immune System,” Science 336, no. 6089 (2012): 1676–1681. 22745423 10.1126/science.1221063 PMC3405774

[2] E. Cai , K. Marchuk , P. Beemiller , et al., “Visualizing Dynamic Microvillar Search and Stabilization During Ligand Detection by T Cells,” Science 356, no. 6338 (2017): 3118, 10.1126/science.aal3118. PMC6364556 28495700

[3] M. M. Davis and P. J. Bjorkman , “T‐Cell Antigen Receptor Genes and T‐Cell Recognition,” Nature 334, no. 6181 (1988): 395–402. 3043226 10.1038/334395a0

[4] G. Oliveira and C. J. Wu , “Dynamics and Specificities of T Cells in Cancer Immunotherapy,” Nature Reviews. Cancer 23, no. 5 (2023): 295–316. 37046001 10.1038/s41568-023-00560-y PMC10773171

[5] R. J. Mallis , K. N. Brazin , J. S. Duke‐Cohan , et al., “Biophysical and Structural Features of αβT‐Cell Receptor Mechanosensing: A Paradigmatic Shift in Understanding T‐Cell Activation,” Immunological Reviews 329, no. 1 (2025): e13432, 10.1111/imr.13432. 39745432 PMC11744257

[6] M. L. Dustin , “Recent Advances in Understanding TCR Signaling: A Synaptic Perspective,” Faculty Reviews 12 (2023): 25. 37900153 10.12703/r/12-25 PMC10608137

[7] S. J. Davis and P. A. van der Merwe , “The Structure and Ligand Interactions of CD2: Implications for T‐Cell Function,” Immunology Today 17, no. 4 (1996): 177–187. 8871350 10.1016/0167-5699(96)80617-7

[8] S. J. Davis and P. A. van der Merwe , “The Kinetic‐Segregation Model: TCR Triggering and Beyond,” Nature Immunology 7, no. 8 (2006): 803–809. 16855606 10.1038/ni1369

[9] H. Xu and D. R. Littman , “A Kinase‐Independent Function of Lck in Potentiating Antigen‐Specific T Cell Activation,” Cell 74, no. 4 (1993): 633–643. 8358792 10.1016/0092-8674(93)90511-n

[10] N. Jiang , J. Huang , L. J. Edwards , et al., “Two‐Stage Cooperative T Cell Receptor‐Peptide Major Histocompatibility Complex‐CD8 Trimolecular Interactions Amplify Antigen Discrimination,” Immunity 34, no. 1 (2011): 13–23, 10.1016/j.immuni.2010.12.017. 21256056 PMC3381515

[11] J. Casas , J. Brzostek , V. I. Zarnitsyna , et al., “Ligand‐Engaged TCR Is Triggered by Lck Not Associated With CD8 Coreceptor,” Nature Communications 5, no. 1 (2014): 5624. 10.1038/ncomms6624 PMC4248239 25427562

[12] A. H. Courtney , W. L. Lo , and A. Weiss , “TCR Signaling: Mechanisms of Initiation and Propagation,” Trends in Biochemical Sciences 43, no. 2 (2018): 108–123. 29269020 10.1016/j.tibs.2017.11.008 PMC5801066

[13] X. Su , J. A. Ditlev , E. Hui , et al., “Phase Separation of Signaling Molecules Promotes T Cell Receptor Signal Transduction,” Science 352, no. 6285 (2016): 595–599, 10.1126/science.aad9964. 27056844 PMC4892427

[14] D. B. McAffee , M. K. O'Dair , J. J. Lin , et al., “Discrete LAT Condensates Encode Antigen Information From Single pMHC:TCR Binding Events,” Nature Communications 13, no. 1 (2022): 7446, 10.1038/s41467-022-35093-9. PMC9718779 36460640

[15] N. M. Edner , G. Carlesso , J. S. Rush , and L. S. K. Walker , “Targeting Co‐Stimulatory Molecules in Autoimmune Disease,” Nature Reviews. Drug Discovery 19, no. 12 (2020): 860–883. 32939077 10.1038/s41573-020-0081-9

[16] K. P. Burke , A. Chaudhri , G. J. Freeman , and A. H. Sharpe , “The B7:CD28 Family and Friends: Unraveling Coinhibitory Interactions,” Immunity 57, no. 2 (2024): 223–244. 38354702 10.1016/j.immuni.2024.01.013 PMC10889489

[17] J. Chin‐Hun Kuo , J. G. Gandhi , R. N. Zia , and M. J. Paszek , “Physical Biology of the Cancer Cell Glycocalyx,” Nature Physics 14, no. 7 (2018): 658–669. 33859716 10.1038/s41567-018-0186-9 PMC8046174

[18] B. Belardi , S. Son , J. H. Felce , M. L. Dustin , and D. A. Fletcher , “Cell‐Cell Interfaces as Specialized Compartments Directing Cell Function,” Nature Reviews. Molecular Cell Biology 21, no. 12 (2020): 750–764. 33093672 10.1038/s41580-020-00298-7

[19] W. R. Brown , A. N. Barclay , C. A. Sunderland , and A. F. Williams , “Identification of a Glycophorin‐Like Molecule at the Cell Surface of Rat Thymocytes,” Nature 289, no. 5797 (1981): 456–460. 6970337 10.1038/289456a0

[20] A. F. Williams and A. N. Barclay , “Glycoprotein Antigens of the Lymphocyte Surface and Their Purification by Antibody Affinity Chromatography,” in Handbook of Experimental Immunology (Blackwell Scientific Publications, 1986), 22.21–22.24.

[21] D. S. Goodsell , L. Autin , and A. J. Olson , “Illustrate: Software for Biomolecular Illustration,” Structure 27, no. 11 (2019): 1716–1720, 10.1016/j.str.2019.08.011. 31519398 PMC6834899

[22] M. A. Lomize , I. D. Pogozheva , H. Joo , H. I. Mosberg , and A. L. Lomize , “OPM Database and PPM Web Server: Resources for Positioning of Proteins in Membranes,” Nucleic Acids Research 40 (2012): D370–D376, 10.1093/nar/gkr703. 21890895 PMC3245162

[23] E. Jenkins , M. Körbel , C. O'Brien‐Ball , et al., “Antigen Discrimination by T Cells Relies on Size‐Constrained Microvillar Contact,” Nature Communications 14, no. 1 (2023): 1611, 10.1038/s41467-023-36855-9. PMC10036606 36959206

[24] A. Gérard , A. P. Cope , C. Kemper , R. Alon , and R. Köchl , “LFA‐1 in T Cell Priming, Differentiation, and Effector Functions,” Trends in Immunology 42, no. 8 (2021): 706–722. 34266767 10.1016/j.it.2021.06.004 PMC10734378

[25] R. A. Fernandes , K. A. Ganzinger , J. C. Tzou , et al., “A Cell Topography‐Based Mechanism for Ligand Discrimination by the T Cell Receptor,” Proceedings of the National Academy of Sciences of the United States of America 116, no. 28 (2019): 14002–14010. 31221762 10.1073/pnas.1817255116 PMC6628812

[26] N. J. Burroughs , Z. Lazic , and P. A. van der Merwe , “Ligand Detection and Discrimination by Spatial Relocalization: A Kinase‐Phosphatase Segregation Model of TCR Activation,” Biophysical Journal 91, no. 5 (2006): 1619–1629. 16751250 10.1529/biophysj.105.080044 PMC1544308

[27] E. Y. Jones , S. J. Davis , A. F. Williams , K. Harlos , and D. I. Stuart , “Crystal Structure at 2.8 Å Resolution of a Soluble Form of the Cell Adhesion Molecule CD2,” Nature 360, no. 6401 (1992): 232–239. 1279440 10.1038/360232a0

[28] D. L. Bodian , E. Y. Jones , K. Harlos , D. I. Stuart , and S. J. Davis , “Crystal Structure of the Extracellular Region of the Human Cell Adhesion Molecule CD2 at 2.5Å Resolution,” Structure 2, no. 8 (1994): 755–766. 7994575 10.1016/s0969-2126(94)00076-x

[29] S. Ikemizu , L. M. Sparks , P. A. van der Merwe , et al., “Crystal Structure of the CD2‐Binding Domain of CD58 (Lymphocyte Function‐Associated Antigen 3) at 1.8‐Å Resolution,” Proceedings of the National Academy of Sciences of the United States of America 96, no. 8 (1999): 4289–4294. 10200255 10.1073/pnas.96.8.4289 PMC16325

[30] E. J. Evans , M. A. A. Castro , R. O'Brien , et al., “Crystal Structure and Binding Properties of the CD2 and CD244 (2B4)‐Binding Protein, CD48,” Journal of Biological Chemistry 281, no. 39 (2006): 29309–29320. 16803907 10.1074/jbc.M601314200

[31] V. T. Chang , R. A. Fernandes , K. A. Ganzinger , et al., “Initiation of T Cell Signaling by CD45 Segregation at “Close Contacts”,” Nature Immunology 17, no. 5 (2016): 574–582. 26998761 10.1038/ni.3392 PMC4839504

[32] L. Sušac , M. T. Vuong , C. Thomas , et al., “Structure of a Fully Assembled Tumor‐Specific T Cell Receptor Ligated by pMHC,” Cell 185, no. 17 (2022): 3201–3213. 35985289 10.1016/j.cell.2022.07.010 PMC9630439

[33] B. S. Gully , J. Ferreira Fernandes , S. D. Gunasinghe , et al., “Structure of a Fully Assembled γδ T Cell Antigen Receptor,” Nature 634, no. 8034 (2024): 729–736. 39146975 10.1038/s41586-024-07920-0 PMC11485255

[34] E. J. Evans , R. M. Esnouf , R. Manso‐Sancho , et al., “Crystal Structure of a Soluble CD28‐Fab Complex,” Nature Immunology 6, no. 3 (2005): 271–279, 10.1038/ni1170. 15696168

[35] C. C. Stamper , Y. Zhang , J. F. Tobin , et al., “Crystal Structure of the B7‐1/CTLA‐4 Complex That Inhibits Human Immune Responses,” Nature 410, no. 6828 (2001): 608–611. 11279502 10.1038/35069118

[36] C. Yu , A. F. P. Sonnen , R. George , et al., “Rigid‐Body Ligand Recognition Drives Cytotoxic T‐Lymphocyte Antigen 4 (CTLA‐4) Receptor Triggering,” Journal of Biological Chemistry 286, no. 8 (2011): 6685–6696. 21156796 10.1074/jbc.M110.182394 PMC3057841

[37] S. Ikemizu , R. J. Gilbert , J. A. Fennelly , et al., “Structure and Dimerization of a Soluble Form of B7‐1,” Immunity 12, no. 1 (2000): 51–60, 10.1016/S1074-7613(00)80158-2. 10661405

[38] X. Cheng , V. Veverka , A. Radhakrishnan , et al., “Structure and Interactions of the Human Programmed Cell Death 1 Receptor,” Journal of Biological Chemistry 288, no. 17 (2013): 11771–11785. 23417675 10.1074/jbc.M112.448126 PMC3636866

[39] A. J. Parodi , “Protein Glucosylation and Its Role in Protein Folding,” Annual Review of Biochemistry 69, no. 1 (2000): 69–93. 10.1146/annurev.biochem.69.1.69 10966453

[40] P. D. Kwong , R. Wyatt , E. Desjardins , et al., “Probability Analysis of Variational Crystallization and Its Application to gp120, the Exterior Envelope Glycoprotein of Type 1 Human Immunodeficiency Virus (HIV‐1),” Journal of Biological Chemistry 274, no. 7 (1999): 4115–4123. 9933605 10.1074/jbc.274.7.4115

[41] J. H. Prestegard , “A Perspective on the PDB's Impact on the Field of Glycobiology,” Journal of Biological Chemistry 296 (2021): 100556. 33744289 10.1016/j.jbc.2021.100556 PMC8058564

[42] S. J. Davis , M. J. Puklavec , D. A. Ashford , et al., “Expression of Soluble Recombinant Glycoproteins With Predefined Glycosylation: Application to the Crystallization of the T‐Cell Glycoprotein CD2,” Protein Engineering, Design & Selection 6, no. 2 (1993): 229–232. 10.1093/protein/6.2.229 8097313

[43] S. J. Davis , E. A. Davies , A. N. Barclay , et al., “Ligand Binding by the Immunoglobulin Superfamily Recognition Molecule CD2 Is Glycosylation‐Independent,” Journal of Biological Chemistry 270, no. 1 (1995): 369–375. 7529232 10.1074/jbc.270.1.369

[44] V. T. Chang , M. Crispin , A. R. Aricescu , et al., “Glycoprotein Structural Genomics: Solving the Glycosylation Problem,” Structure 15, no. 3 (2007): 267–273. 17355862 10.1016/j.str.2007.01.011 PMC1885966

[45] P. A. van der Merwe and S. J. Davis , “Molecular Interactions Mediating T Cell Antigen Recognition,” Annual Review of Immunology 21, no. 1 (2003): 659–684. 10.1146/annurev.immunol.21.120601.141036 12615890

[46] S. J. Davis , S. Ikemizu , E. J. Evans , L. Fugger , T. R. Bakker , and P. A. van der Merwe , “The Nature of Molecular Recognition by T Cells,” Nature Immunology 4, no. 3 (2003): 217–224. 12605231 10.1038/ni0303-217

[47] M. E. McNerney and V. Kumar , “The CD2 Family of Natural Killer Cell Receptors,” Current Topics in Microbiology and Immunology 298 (2006): 91–120. 16323413 10.1007/3-540-27743-9_5

[48] T. Hünig , “The Cell Surface Molecule Recognized by the Erythrocyte Receptor of T Lymphocytes. Identification and Partial Characterization Using a Monoclonal Antibody,” Journal of Experimental Medicine 162, no. 3 (1985): 890–901. 2411842 10.1084/jem.162.3.890 PMC2187812

[49] P. Selvaraj , M. L. Plunkett , M. Dustin , M. E. Sanders , S. Shaw , and T. A. Springer , “The T Lymphocyte Glycoprotein CD2 Binds the Cell Surface Ligand LFA‐3,” Nature 326, no. 6111 (1987): 400–403. 2951597 10.1038/326400a0

[50] P. A. van der Merwe , M. H. Brown , S. J. Davis , and A. N. Barclay , “Affinity and Kinetic Analysis of the Interaction of the Cell Adhesion Molecules Rat CD2 and CD48,” EMBO Journal 12, no. 13 (1993): 4945–4954. 7903240 10.1002/j.1460-2075.1993.tb06188.x PMC413755

[51] P. A. van der Merwe and A. N. Barclay , “Transient Intercellular Adhesion: The Importance of Weak Protein‐Protein Interactions,” Trends in Biochemical Sciences 19, no. 9 (1994): 354–358. 7985226 10.1016/0968-0004(94)90109-0

[52] Y. Zhang , Q. Liu , S. Yang , and Q. Liao , “CD58 Immunobiology at a Glance,” Frontiers in Immunology 12 (2021): 705260. 34168659 10.3389/fimmu.2021.705260 PMC8218816

[53] N. Killeen , S. G. Stuart , and D. R. Littman , “Development and Function of T Cells in Mice With a Disrupted CD2 Gene,” EMBO Journal 11, no. 12 (1992): 4329–4336. 1358605 10.1002/j.1460-2075.1992.tb05532.x PMC557006

[54] M. F. Bachmann , M. Barner , and M. Kopf , “CD2 Sets Quantitative Thresholds in T Cell Activation,” Journal of Experimental Medicine 190, no. 10 (1999): 1383–1392. 10562314 10.1084/jem.190.10.1383 PMC2195700

[55] K. Kato , M. Koyanagi , H. Okada , et al., “CD48 Is a Counter‐Receptor for Mouse CD2 and Is Involved in T Cell Activation,” Journal of Experimental Medicine 176, no. 5 (1992): 1241–1249. 1383383 10.1084/jem.176.5.1241 PMC2119417

[56] M. H. Brown , K. Boles , P. A. van der Merwe , V. Kumar , P. A. Mathew , and A. N. Barclay , “2B4, the Natural Killer and T Cell Immunoglobulin Superfamily Surface Protein, Is a Ligand for CD48,” Journal of Experimental Medicine 188, no. 11 (1998): 2083–2090. 9841922 10.1084/jem.188.11.2083 PMC2212392

[57] B. A. Garni‐Wagner , A. Purohit , P. A. Mathew , M. Bennett , and V. Kumar , “A Novel Function‐Associated Molecule Related to Non‐MHC‐Restricted Cytotoxicity Mediated by Activated Natural Killer Cells and T Cells,” Journal of Immunology 151, no. 1 (1993): 60–70. 8326140

[58] A. F. Williams , A. N. Barclay , S. J. Clark , D. J. Paterson , and A. C. Willis , “Similarities in Sequences and Cellular Expression Between Rat CD2 and CD4 Antigens,” Journal of Experimental Medicine 165, no. 2 (1987): 368–380. 3102667 10.1084/jem.165.2.368 PMC2188524

[59] A. S. Tavernor , J. H. Kydd , D. L. Bodian , et al., “Expression Cloning of an Equine T‐Lymphocyte Glycoprotein CD2 cDNA. Structure‐Based Analysis of Conserved Sequence Elements: Structure‐Based Analysis of Conserved Sequence Elements,” European Journal of Biochemistry 219, no. 3 (1994): 969–976. 7906650 10.1111/j.1432-1033.1994.tb18579.x

[60] J. H. Wang , A. Smolyar , K. Tan , et al., “Structure of a Heterophilic Adhesion Complex Between the Human CD2 and CD58 (LFA‐3) Counterreceptors,” Cell 97, no. 6 (1999): 791–803. 10380930 10.1016/s0092-8674(00)80790-4

[61] Y. X. Tsai , N. E. Chang , K. Reuter , et al., “Rapid Simulation of Glycoprotein Structures by Grafting and Steric Exclusion of Glycan Conformer Libraries,” Cell 187, no. 5 (2024): 1296–1311.e26. 38428397 10.1016/j.cell.2024.01.034

[62] A. Peterson and B. Seed , “Monoclonal Antibody and Ligand Binding Sites of the T Cell Erythrocyte Receptor (CD2),” Nature 329, no. 6142 (1987): 842–846. 2444890 10.1038/329842a0

[63] C. Somoza , P. C. Driscoll , J. G. Cyster , and A. F. Williams , “Mutational Analysis of the CD2/CD58 Interaction: The Binding Site for CD58 Lies on One Face of the First Domain of Human CD2,” Journal of Experimental Medicine 178, no. 2 (1993): 549–558. 7688025 10.1084/jem.178.2.549 PMC2191138

[64] A. R. Arulanandam , J. M. Withka , D. F. Wyss , et al., “The CD58 (LFA‐3) Binding Site Is a Localized and Highly Charged Surface Area on the AGFCC'C Face of the Human CD2 Adhesion Domain,” Proceedings of the National Academy of Sciences of the United States of America 90, no. 24 (1993): 11613–11617. 7505442 10.1073/pnas.90.24.11613 PMC48034

[65] S. J. Davis , E. A. Davies , M. G. Tucknott , E. Y. Jones , and P. A. van der Merwe , “The Role of Charged Residues Mediating Low Affinity Protein‐Protein Recognition at the Cell Surface by CD2,” Proceedings of the National Academy of Sciences of the United States of America 95, no. 10 (1998): 5490–5494. 9576909 10.1073/pnas.95.10.5490 PMC20404

[66] B. Honig and A. Nicholls , “Classical Electrostatics in Biology and Chemistry,” Science 268, no. 5214 (1995): 1144–1149. 7761829 10.1126/science.7761829

[67] J. Janin and C. Chothia , “The Structure of Protein‐Protein Recognition Sites,” Journal of Biological Chemistry 265, no. 27 (1990): 16027–16030. 2204619

[68] A. R. Arulanandam , A. Kister , M. J. McGregor , D. F. Wyss , G. Wagner , and E. L. Reinherz , “Interaction Between Human CD2 and CD58 Involves the Major Beta Sheet Surface of Each of Their Respective Adhesion Domains,” Journal of Experimental Medicine 180, no. 5 (1994): 1861–1871. 7525842 10.1084/jem.180.5.1861 PMC2191747

[69] L. Osborn , E. S. Day , G. T. Miller , et al., “Amino Acid Residues Required for Binding of Lymphocyte Function‐Associated Antigen 3 (CD58) to Its Counter‐Receptor CD2,” Journal of Experimental Medicine 181, no. 1 (1995): 429–434. 7528777 10.1084/jem.181.1.429 PMC2191834

[70] E. H. Fischer , H. Charbonneau , D. E. Cool , and N. K. Tonks , “Tyrosine phosphatases and their possible interplay with tyrosine kinases,” Ciba Found Symp 164 (1992): 132–140, 10.1002/9780470514207. 1395930

[71] J. T. Pingel and M. L. Thomas , “Evidence That the Leukocyte‐Common Antigen Is Required for Antigen‐Induced T Lymphocyte Proliferation,” Cell 58, no. 6 (1989): 1055–1065. 2550143 10.1016/0092-8674(89)90504-7 PMC7127598

[72] D. M. Desai , J. Sap , O. Silvennoinen , J. Schlessinger , and A. Weiss , “The Catalytic Activity of the CD45 Membrane‐Proximal Phosphatase Domain Is Required for TCR Signaling and Regulation,” EMBO Journal 13, no. 17 (1994): 4002–4010. 8076596 10.1002/j.1460-2075.1994.tb06716.x PMC395320

[73] M. L. Hermiston , Z. Xu , and A. Weiss , “CD45: A Critical Regulator of Signaling Thresholds in Immune Cells,” Annual Review of Immunology 21, no. 1 (2003): 107–137. 10.1146/annurev.immunol.21.120601.140946 12414720

[74] A. R. Aricescu , C. Siebold , K. Choudhuri , et al., “Structure of a Tyrosine Phosphatase Adhesive Interaction Reveals a Spacer‐Clamp Mechanism,” Science 317, no. 5842 (2007): 1217–1220. 17761881 10.1126/science.1144646

[75] C. H. Coles , N. Mitakidis , P. Zhang , et al., “Structural Basis for Extracellular cis and Trans RPTPσ Signal Competition in Synaptogenesis,” Nature Communications 5, no. 1 (2014): 5209. 10.1038/ncomms6209 PMC4239663 25385546

[76] J. R. James , J. McColl , M. I. Oliveira , et al., “The T Cell Receptor Triggering Apparatus Is Composed of Monovalent or Monomeric Proteins,” Journal of Biological Chemistry 286, no. 37 (2011): 31993–32001. 21757710 10.1074/jbc.M111.219212 PMC3173209

[77] T. Nogi , N. Yasui , E. Mihara , et al., “Structural Basis for Semaphorin Signalling Through the Plexin Receptor,” Nature 467, no. 7319 (2010): 1123–1127. 20881961 10.1038/nature09473

[78] N. Jentoft , “Why Are Proteins O‐Glycosylated?,” Trends in Biochemical Sciences 15, no. 8 (1990): 291–294. 2204153 10.1016/0968-0004(90)90014-3

[79] A. Morath and W. W. Schamel , “αβ and γδ T Cell Receptors: Similar but Different,” Journal of Leukocyte Biology 107, no. 6 (2020): 1045–1055. 31994778 10.1002/JLB.2MR1219-233R

[80] K. C. Garcia , M. Degano , R. L. Stanfield , et al., “An Alphabeta T Cell Receptor Structure at 2.5 A and Its Orientation in the TCR‐MHC Complex,” Science 274, no. 5285 (1996): 209–219, 10.1126/science.274.5285.209. 8824178

[81] D. N. Garboczi , P. Ghosh , U. Utz , Q. R. Fan , W. E. Biddison , and D. C. Wiley , “Structure of the Complex Between Human T‐Cell Receptor, Viral Peptide and HLA‐A2,” Nature 384, no. 6605 (1996): 134–141. 8906788 10.1038/384134a0

[82] Z. J. Sun , K. S. Kim , G. Wagner , and E. L. Reinherz , “Mechanisms Contributing to T Cell Receptor Signaling and Assembly Revealed by the Solution Structure of an Ectodomain Fragment of the CD3 Epsilon Gamma Heterodimer,” Cell 105, no. 7 (2001): 913–923. 11439187 10.1016/s0092-8674(01)00395-6

[83] K. L. Arnett , S. C. Harrison , and D. C. Wiley , “Crystal Structure of a Human CD3‐Epsilon/delta Dimer in Complex With a UCHT1 Single‐Chain Antibody Fragment,” Proceedings of the National Academy of Sciences of the United States of America 101, no. 46 (2004): 16268–16273. 15534202 10.1073/pnas.0407359101 PMC528977

[84] L. Kjer‐Nielsen , M. A. Dunstone , L. Kostenko , et al., “Crystal Structure of the Human T Cell Receptor CD3 Epsilon Gamma Heterodimer Complexed to the Therapeutic mAb OKT3,” Proceedings of the National Academy of Sciences of the United States of America 101, no. 20 (2004): 7675–7680. 15136729 10.1073/pnas.0402295101 PMC419665

[85] M. E. Call , J. R. Schnell , C. Xu , R. A. Lutz , J. J. Chou , and K. W. Wucherpfennig , “The Structure of the Zetazeta Transmembrane Dimer Reveals Features Essential for Its Assembly With the T Cell Receptor,” Cell 127, no. 2 (2006): 355–368. 17055436 10.1016/j.cell.2006.08.044 PMC3466601

[86] M. G. Rudolph , R. L. Stanfield , and I. A. Wilson , “How TCRs Bind MHCs, Peptides, and Coreceptors,” Annual Review of Immunology 24, no. 1 (2006): 419–466. 10.1146/annurev.immunol.23.021704.115658 16551255

[87] D. Dong , L. Zheng , J. Lin , et al., “Structural Basis of Assembly of the Human T Cell Receptor‐CD3 Complex,” Nature 573, no. 7775 (2019): 546–552. 31461748 10.1038/s41586-019-1537-0

[88] Y. Chen , Y. Zhu , X. Li , et al., “Cholesterol Inhibits TCR Signaling by Directly Restricting TCR‐CD3 Core Tunnel Motility,” Molecular Cell 82, no. 7 (2022): 1278–1287.e5. 35271814 10.1016/j.molcel.2022.02.017

[89] N. Liddy , “Molecular Engineering of High Affinity T‐Cell Receptors for Bispecific Therapeutics. Phd,” 2013 Cardiff University, https://orca.cardiff.ac.uk/id/eprint/47271/.

[90] K. Saotome , D. Dudgeon , K. Colotti , et al., “Structural Analysis of Cancer‐Relevant TCR‐CD3 and Peptide‐MHC Complexes by cryoEM,” Nature Communications 14, no. 1 (2023): 2401. 10.1038/s41467-023-37532-7 PMC10132440 37100770

[91] Y. Hu , Q. Hu , Y. Li , et al., “γδ T Cells: Origin and Fate, Subsets, Diseases and Immunotherapy,” Signal Transduction and Targeted Therapy 8, no. 1 (2023): 434. 37989744 10.1038/s41392-023-01653-8 PMC10663641

[92] J. Rossjohn , S. Gras , J. J. Miles , S. J. Turner , D. I. Godfrey , and J. McCluskey , “T Cell Antigen Receptor Recognition of Antigen‐Presenting Molecules,” Annual Review of Immunology 33 (2015): 169–200. 10.1146/annurev-immunol-032414-112334 25493333

[93] T. J. Allison , C. C. Winter , J. J. Fournié , M. Bonneville , and D. N. Garboczi , “Structure of a Human Gammadelta T‐Cell Antigen Receptor,” Nature 411, no. 6839 (2001): 820–824. 11459064 10.1038/35081115

[94] W. Hwang , R. J. Mallis , M. J. Lang , and E. L. Reinherz , “The αβTCR Mechanosensor Exploits Dynamic Ectodomain Allostery to Optimize Its Ligand Recognition Site,” Proceedings of the National Academy of Sciences of the United States of America 117, no. 35 (2020): 21336–21345. 32796106 10.1073/pnas.2005899117 PMC7474670

[95] P. G. Pelicci , M. Subar , A. Weiss , R. Dalla‐Favera , and D. R. Littman , “Molecular Diversity of the Human T‐Gamma Constant Region Genes,” Science 237, no. 4818 (1987): 1051–1055. 3112943 10.1126/science.3112943

[96] M. P. Lefranc , A. Forster , and T. H. Rabbitts , “Genetic Polymorphism and Exon Changes of the Constant Regions of the Human T‐Cell Rearranging Gene Gamma,” Proceedings of the National Academy of Sciences of the United States of America 83, no. 24 (1986): 9596–9600. 2879283 10.1073/pnas.83.24.9596 PMC387187

[97] W. Xin , B. Huang , X. Chi , et al., “Structures of Human γδ T Cell Receptor‐CD3 Complex,” Nature 630, no. 8015 (2024): 222–229. 38657677 10.1038/s41586-024-07439-4 PMC11153141

[98] M. Hoque , J. B. Grigg , T. Ramlall , et al., “Structural Characterization of Two γδ TCR/CD3 Complexes,” Nature Communications 16, no. 1 (2025): 318, 10.1038/s41467-024-55467-5. PMC11697310 39747888

[99] J. Le Nours , N. A. Gherardin , S. H. Ramarathinam , et al., “A Class of γδ T Cell Receptors Recognize the Underside of the Antigen‐Presenting Molecule MR1,” Science 366, no. 6472 (2019): 1522–1527. 31857486 10.1126/science.aav3900

[100] M. T. Rice , A. von Borstel , P. Chevour , et al., “Recognition of the Antigen‐Presenting Molecule MR1 by a Vδ3+ γδ T Cell Receptor,” Proceedings of the National Academy of Sciences of the United States of America 118, no. 49 (2021): e2110288118. 34845016 10.1073/pnas.2110288118 PMC8694053

[101] S. M. Hayes and P. E. Love , “Distinct Structure and Signaling Potential of the Gamma Delta TCR Complex,” Immunity 16, no. 6 (2002): 827–838. 12121664 10.1016/s1074-7613(02)00320-5

[102] G. M. Siegers , M. Swamy , E. Fernández‐Malavé , et al., “Different Composition of the Human and the Mouse Gammadelta T Cell Receptor Explains Different Phenotypes of CD3gamma and CD3delta Immunodeficiencies,” Journal of Experimental Medicine 204, no. 11 (2007): 2537–2544. 17923503 10.1084/jem.20070782 PMC2118495

[103] C. E. Rudd , A. Taylor , and H. Schneider , “CD28 and CTLA‐4 Coreceptor Expression and Signal Transduction,” Immunological Reviews 229, no. 1 (2009): 12–26. 19426212 10.1111/j.1600-065X.2009.00770.x PMC4186963

[104] J. R. James , M. I. Oliveira , A. M. Carmo , A. Iaboni , and S. J. Davis , “A Rigorous Experimental Framework for Detecting Protein Oligomerization Using Bioluminescence Resonance Energy Transfer,” Nature Methods 3, no. 12 (2006): 1001–1006. 17086179 10.1038/nmeth978

[105] S. Bhatia , M. Edidin , S. C. Almo , and S. G. Nathenson , “Different cell surface oligomeric states of B7‐1 and B7‐2: implications for signaling,” Proc Natl Acad Sci U S A 102, no. 43 (2005): 15569–15574, 10.1073/pnas.0507257102. 16221763 PMC1266120

[106] D. M. Sansom , “CD28, CTLA‐4 and Their Ligands: Who Does What and to Whom?,” Immunology 101, no. 2 (2000): 169–177. 11012769 10.1046/j.1365-2567.2000.00121.x PMC2327073

[107] A. V. Collins , D. W. Brodie , R. J. C. Gilbert , et al., “The Interaction Properties of Costimulatory Molecules Revisited,” Immunity 17, no. 2 (2002): 201–210. 12196291 10.1016/s1074-7613(02)00362-x

[108] P. Sharma , S. Goswami , D. Raychaudhuri , et al., “Immune Checkpoint Therapy‐Current Perspectives and Future Directions,” Cell 186, no. 8 (2023): 1652–1669. 37059068 10.1016/j.cell.2023.03.006

[109] E. A. Philips , J. Liu , A. Kvalvaag , et al., “Transmembrane Domain‐Driven PD‐1 Dimers Mediate T Cell Inhibition,” Science Immunology 9, no. 93 (2024): eade6256, 10.1126/sciimmunol.ade6256. 38457513 PMC11166110

[110] A. H. Lippert , C. Paluch , M. Gaglioni , et al., “Antibody Agonists Trigger Immune Receptor Signaling Through Local Exclusion of Receptor‐Type Protein Tyrosine Phosphatases,” Immunity 57, no. 2 (2024): 256–270. 38354703 10.1016/j.immuni.2024.01.007 PMC7618792

[111] D. M. Sansom and L. S. K. Walker , “The Role of CD28 and Cytotoxic T‐Lymphocyte Antigen‐4 (CTLA‐4) in Regulatory T‐Cell Biology,” Immunological Reviews 212, no. 1 (2006): 131–148. 16903911 10.1111/j.0105-2896.2006.00419.x

[112] F. Lühder , Y. Huang , K. M. Dennehy , et al., “Topological Requirements and Signaling Properties of T Cell‐Activating, Anti‐CD28 Antibody Superagonists,” Journal of Experimental Medicine 197, no. 8 (2003): 955–966, 10.1084/jem.20021024. 12707299 PMC2193880

[113] O. S. Qureshi , Y. Zheng , K. Nakamura , et al., “Trans‐Endocytosis of CD80 and CD86: A Molecular Basis for the Cell‐Extrinsic Function of CTLA‐4,” Science 332, no. 6029 (2011): 600–603, 10.1126/science.1202947. 21474713 PMC3198051

[114] J. C. Schwartz , X. Zhang , A. A. Fedorov , S. G. Nathenson , and S. C. Almo , “Structural Basis for Co‐Stimulation by the Human CTLA‐4/B7‐2 Complex,” Nature 410, no. 6828 (2001): 604–608. 11279501 10.1038/35069112

[115] M. C. Lawrence and P. M. Colman , “Shape Complementarity at Protein/Protein Interfaces,” Journal of Molecular Biology 234, no. 4 (1993): 946–950. 8263940 10.1006/jmbi.1993.1648

[116] P. A. van der Merwe , D. L. Bodian , S. Daenke , P. Linsley , and S. J. Davis , “CD80 (B7‐1) Binds Both CD28 and CTLA‐4 With a Low Affinity and Very Fast Kinetics,” Journal of Experimental Medicine 185, no. 3 (1997): 393–403. 9053440 10.1084/jem.185.3.393 PMC2196039

[117] X. Xu , B. Hou , A. Fulzele , et al., “PD‐1 and BTLA Regulate T Cell Signaling Differentially and Only Partially Through SHP1 and SHP2,” Journal of Cell Biology 219, no. 6 (2020): 5085, 10.1083/jcb.201905085. PMC7265324 32437509

[118] K. M. Zak , R. Kitel , S. Przetocka , et al., “Structure of the Complex of Human Programmed Death 1, PD‐1, and Its Ligand PD‐L1,” Structure 23, no. 12 (2015): 2341–2348, 10.1016/j.str.2015.09.010. 26602187 PMC4752817

[119] D. Y. W. Lin , Y. Tanaka , M. Iwasaki , et al., “The PD‐1/PD‐L1 Complex Resembles the Antigen‐Binding Fv Domains of Antibodies and T Cell Receptors,” Proceedings of the National Academy of Sciences of the United States of America 105, no. 8 (2008): 3011–3016. 18287011 10.1073/pnas.0712278105 PMC2268576

[120] O. Dushek , J. Goyette , and P. A. van der Merwe , “Non‐Catalytic Tyrosine‐Phosphorylated Receptors,” Immunological Reviews 250, no. 1 (2012): 258–276. 23046135 10.1111/imr.12008

[121] E. M. Schmid , M. H. Bakalar , K. Choudhuri , et al., “Size‐Dependent Protein Segregation at Membrane Interfaces,” Nature Physics 12, no. 7 (2016): 704–711. 27980602 10.1038/nphys3678 PMC5152624

[122] D. M. Rothstein , H. Saito , M. Streuli , S. F. Schlossman , and C. Morimoto , “The Alternative Splicing of the CD45 Tyrosine Phosphatase Is Controlled by Negative Regulatory Trans‐Acting Splicing Factors,” Journal of Biological Chemistry 267, no. 10 (1992): 7139–7147. 1532394

[123] P. Zareie , C. Szeto , C. Farenc , et al., “Canonical T Cell Receptor Docking on Peptide‐MHC Is Essential for T Cell Signaling,” Science 372, no. 6546 (2021): eabe9124, 10.1126/science.abe9124. 34083463

[124] R. J. Mallis , J. S. Duke‐Cohan , D. K. Das , et al., “Molecular Design of the γδT Cell Receptor Ectodomain Encodes Biologically Fit Ligand Recognition in the Absence of Mechanosensing,” Proceedings of the National Academy of Sciences of the United States of America 118, no. 26 (2021): e2023050118. 34172580 10.1073/pnas.2023050118 PMC8256041

[125] K. Y. Chen , E. Jenkins , M. Körbel , et al., “Trapping or Slowing the Diffusion of T Cell Receptors at Close Contacts Initiates T Cell Signaling,” Proceedings of the National Academy of Sciences of the United States of America 118, no. 39 (2021): e2024250118. 34526387 10.1073/pnas.2024250118 PMC8488633

[126] R. Q. Notti , F. Yi , S. Heissel , et al., “The Resting and Ligand‐Bound States of the Membrane‐Embedded Human T‐Cell Receptor‐CD3 Complex,” bioRxivorg 2024 (2024): 54360, 10.1101/2023.08.22.554360. PMC12708868 41402338

